# Exploring the mechanism of artificial selection signature in Chinese indigenous pigs by leveraging multiple bioinformatics database tools

**DOI:** 10.1186/s12864-023-09848-7

**Published:** 2023-12-05

**Authors:** Xueyan Feng, Shuqi Diao, Yuqiang Liu, Zhiting Xu, Guangzhen Li, Ye Ma, Zhanqin Su, Xiaohong Liu, Jiaqi Li, Zhe Zhang

**Affiliations:** 1 State Key Laboratory of Swine and Poultry Breeding Industry, Guangdong Provincial Key Lab of Agro-Animal Genomics and Molecular Breeding, College of Animal Science, South China Agricultural University, Guangzhou, 510642 China; 2 State Key Laboratory of Biocontrol, School of Life Sciences, Sun Yat-Sen University, Guangzhou, 510275 China

**Keywords:** Chinese indigenous pigs, Artificial selection, Genetic diversity, Bioinformatics databases

## Abstract

**Background:**

Chinese indigenous pigs in Yunnan exhibit considerable phenotypic diversity, but their population structure and the biological interpretation of signatures of artificial selection require further investigation. To uncover population genetic diversity, migration events, and artificial selection signatures in Chinese domestic pigs, we sampled 111 Yunnan pigs from four breeds in Yunnan which is considered to be one of the centres of livestock domestication in China, and genotyped them using Illumina Porcine SNP60K BeadChip. We then leveraged multiple bioinformatics database tools to further investigate the signatures and associated complex traits.

**Results:**

Population structure and migration analyses showed that Diannanxiaoer pigs had different genetic backgrounds from other Yunnan pigs, and Gaoligongshan may undergone the migration events from Baoshan and Saba pigs. Intriguingly, we identified a possible common target of sharing artificial selection on a 265.09 kb region on chromosome 5 in Yunnan indigenous pigs, and the genes on this region were associated with cardiovascular and immune systems. We also detected several candidate genes correlated with dietary adaptation, body size (e.g., *PASCIN1*, *GRM4*, *ITPR2*), and reproductive performance. In addition, the breed-sharing gene *MMP16* was identified to be a human-mediated gene. Multiple lines of evidence at the mammalian genome, transcriptome, and phenome levels further supported the evidence for the causality between *MMP16* variants and the metabolic diseases, brain development, and cartilage tissues in Chinese pigs. Our results suggested that the suppression of *MMP16* would directly lead to inactivity and insensitivity of neuronal activity and skeletal development in Chinese indigenous pigs.

**Conclusion:**

In this study, the population genetic analyses and identification of artificial selection signatures of Yunnan indigenous pigs help to build an understanding of the effect of human-mediated selection mechanisms on phenotypic traits in Chinese indigenous pigs. Further studies are needed to fully characterize the process of human-mediated genes and biological mechanisms.

**Supplementary Information:**

The online version contains supplementary material available at 10.1186/s12864-023-09848-7.

## Introduction

Pigs (*Sus scrofa*) were domesticated separately from the local wild boar in China and Anatolia about 10,000 years ago [1–3] and gradually formed different pig breeds in several Eurasian regions [3–5], about one third of which can be found in China [4, 6]. Yunnan, southwestern China, which is covered by the Mekong region, is one of the several proposed Asian domestication sites of pigs [7, 8] and has been proposed as a center of domestication of livestock and poultry in China [9–11]. Bred in extremely diverse geographical environments and ethnic minority cultures, many exclusive Yunnan pig breeds have excellent characteristics such as tolerance to rough feeding, good adaptability, and resistance to diseases and parasites [12].

As one of the first domesticated species [13], pigs have undergone parallel evolution with humans in the history of early hunting-gathering and the more recent change in living environment, feeding habits, and breeding requirements from agrarian societies to modern cities. The human-mediated domestication results in striking differences in appearance, growth, reproduction, behavior, and central nervous system [14, 15], such as less aggressive behavior, reduced responsiveness, and changes in taste perception between domesticated animals and their wild ancestors. For example, detection of selection signatures and analysis of epigenomic regulatory elements analyses in European commercial pig breeds, which have undergone strong artificial selection suggested that the domestication process affected brain functions associated with memory and learning [16], and there was a strong difference between the fear-associated aggressive behaviors of the wild population and the tame behavior of the domestic populations [17]. As a promising model animal for identifying genetic variation during artificial selection and understanding its biological impact on mammalian traits and disease susceptibility, the pig has been used as a human biomedical model to provide insights into complex mechanisms of metabolism [18, 19] and immunology [20–22], reproductive function [23, 24], and neurological disease [25–27].

Genome-wide scanning to identify selection signatures has been widely applied to further explore the phenotypic diversity of famous European commercial pig breeds [28–30] (e.g. Duroc, Landrace) and excellent Chinese pig breeds [31–33] (e.g., Laiwu, Taihu pigs). Recent studies of Yunnan pigs are more focused on their body size, meat quality, crossbreeding utilization, microbiota, and viral pathology [34–36], while studies on the genetic structure and selection signatures of breed populations in the context of adaptive evolution are relatively lacking. As a result of long-term domestication under complex topography, abundant rainfall and light, and the multi-ethnic eating and customs in Yunnan, we proposed that processing whole-genome selection signature analysis on Yunnan indigenous pig breeds to investigate different selected footprints on the genome across breeds would advance valuable insights related to artificial selection and economic traits in Chinese indigenous pigs.

Nowadays, more and more large-scale international projects are established to systematically decipher genetic regulatory mechanisms and develop several comprehensive public resources of genetic regulatory variants in different mammalian species, e.g., the Human Phenome-Wide Association Analysis (PheWAS) atlas [37, 38], the Pig Genotype-Tissue Expression (PigGTEx) project [39, 40], the International Mouse Phenotyping Consortium (IMPC) portal [41, 42], Human Protein Atlas (HPA) portal [43, 44]. While these open access resources facilitate comprehensive discovery of biology, they also provide researchers with many extensive interactive public bioinformatics database tools to further understand the complex interaction among selective genetic variants, variation in phenotypes, and biological functions. Leveraging the extensive public resources, we deciphered a dozen causative variants underlying the mechanism of artificial selection signatures during the domestication progress of Chinese indigenous pigs, such as brain development, fat deposition, reproduction, and meat growth. The work presented here demonstrates the utility of extensive bioinformatics database tools in providing new insights into the genetic mechanism of artificial selective variation and the genetic diversity of germplasm traits.

## Materials and methods

### Sample collection and genotype processing

In this study, a total of 111 Yunnan indigenous pigs from four breeds and 33 Asian wild boars (WBA) were included for analysis (Table 1). For the Yunnan indigenous pig samples, the populations of 28 Diannanxiaoer (DNXE) and 28 Gaoligongshan (GLGS) pigs were genotyped for 61,565 SNPs on the Illumina Porcine SNP60K BeadChip [45]. We recruited the raw genotype data of 32 Baoshan (BS) and 23 Saba (SB) pigs [46] and 33 WBA [5] from previous studies. To obtain reliable samples, we sampled the ear tissues of DNXE and GLGS which were selected to maximize the genetic representation within each breed according to available records for genotyping with consideration for animal welfare. Genomic DNA was extracted using the Tissue DNA Kit (Omega Bio-tek, Norcross, GA, USA).

**Table 1:** Sample sizes and locations of Asian wild boars and four Yunnan indigenous pig breeds

No.	Breed	Abbreviation	Sample size	Average altitude	Sample site (province/ autonomous region)
1	Baoshan	BS	32	1669 m	Baoshan City, Yunnan Province
2	Diannanxiaoer	DNXE	28	1095 m	Xishuangbanna Dai Autonomous Prefecture, Yunnan province
3	Gaoligongshan	GLGS	28	2343 m	Nuking Prefecture, Yunnan province
4	Saba	SB	23	1920 m	Chuxiong Prefecture, Yunnan Province
5	Asian wild boars	WBA	33	/	Korea/ North of China /Russia and East of Russia
	**SUM.**		**144**		

Raw genotypes with 53,305 SNPs were obtained based on the *Sus Scrofa* 11.1 genome, after merging the genotypes and eliminating the SNPs with unknown location using PLINK v1.90 [47]. We conducted the quality control procedures as follows: (1) filtered out individuals with a call rate less than 0.90; (2) removed SNPs on sex chromosomes; (3) discarded SNPs with a call rate less than 0.90; (4) removed SNPs with a minor allele frequency less than 0.01. A final set of 144 individuals and 44,985 SNPs were retained for the subsequent analysis.

#### New-added samples and genotypes

To show the phylogenetic position of the Chinese indigenous pig populations in the context of a broad panel of breeds, we downloaded raw genotype data from Yang et al. [5] of three internationally well-known European commercial pig breeds (Large White, Duroc, and Landrace pigs) based on Illumina Porcine SNP60K BeadChip. The raw genotypes with 61,772 SNPs on the *Sus Scrofa* 10.2 genome of 76 Large White pigs (LWT), 130 Landrace pigs (LDR), and 79 Duroc pigs (DUR). We first conducted the same genotype quality control process and Linkage disequilibrium filter. After quality control, a total of 56,646 SNPs were maintained based on the *Sus Scrofa* 11.1 genome to perform PCA (“--pca 10”) for single breed population to check the overall structure of these samples (Fig. S1a) and initially exclude the outliers. And then 59 DUR (lineage 1, 3, 4), 40 LWT (lineage 2, 3), and 70 LDR (lineage 1–4) were kept for further selection of key individuals (Fig. S1b-d).

#### Key individuals’ selection

To maintain a balance with the sample size in this study, we planned to select 30 DUR, LWT, and LDR individuals separately. To ensure that the key individuals are maximally representative of the group, the definition of the key individual of the group is that the key individual needs to be maximally related to other individuals in the population. In this study, 30 key individuals in DUR, LWT, and LDR were separately screened (Fig. S1e) according to the Maximizing expected genetic relationship (MEGR) method proposed by Druet et al. [48]. We constructed the molecular kinship matrix (G-matrix) of the population by using the method proposed by VanRaden [49] based on SNP markers. This procedure was performed using the GCTA v1.94.0 program [50] and the command was: --make-grm --make-grm-alg 0.

After merging the genotypes of 144 Yunnan indigenous pigs, Asian wild boars, and 90 new-added European commercial pigs and eliminating those SNPs with unknown location, raw genotypes with 53,287 SNPs was maintained based on the *Sus Scrofa* 11.1 genome. After the same quality control process, a final set of 234 individuals and 50,156 SNPs were retained.

### Population genetic analyses of Yunnan indigenous pigs

To investigate the population genetic structures of Yunnan indigenous pigs, we conducted the phylogenetic Neighbor-Joining (NJ) tree analysis, principal component analysis (PCA), and admixture analysis based on the 34,459 LD pruned-in SNPs via PLINK v1.90 [47] by the command: --indep-pairwise 50 5 0.5 with pairwise *r*^*2*^ threshold of 0.5. We constructed the NJ tree using a pairwise identity by state (IBS) matrix obtained by PLINK v1.90 [47] and visualized via MEGA v11 [51] and iTOL v5.0 [52]. We performed PCA using PLINK v1.90 [47] software and evaluated the population genetic structure using ADMIXTURE v1.3.0 [53] program considering genetic clusters (*K)* range 2 to 6.

To evaluate the genetic diversity of Yunnan indigenous pigs, we estimated the expected and observed heterozygosity (He, Ho), *F*_ST_ genetic distance, and linkage disequilibrium (LD) decay. We estimated He and Ho using PLINK v1.90 [47] and visualized the results via R package ggplot2 v3.3.6 [54]. We constructed the maximum likelihood (ML) tree via MEGA v1 1[51] based on the genome-wide pairwise *F*_ST_ matrices which were estimated using the SMARTPCA program [55] and computed *F*_ST_ values for each SNP as described by Karlsson et al. [56]. We performed genome-wide LD decay in the distance of 10 Mb using PopLDdecay v3.30 [57] (https://github.com/BGI-shenzhen/PopLDdecay). We visualized the results of population genetic structure and genome-wide LD decay via R v4.1.0 [58].

We also conducted the population genetic analyses which included PCA, NJ tree reconstruction analyses, and Admixture ancestry models with *K* ranging from 2 to 9 based on 33,304 LD pruned-in SNPs of 234 individuals which consist of four Chinese Yunnan indigenous pig breeds, Asian wild boars and three European commercial pig breeds via PLINK v1.90 [47].

### Migration events and genetic introgression analyses

To infer migration events among the five breeds, we constructed an ML tree with the root group WBA and 1000 SNPs per window, and tested 1–5 migration events and corresponding residuals using TreeMix v1.13 [59]. Then we further investigated the admixture by performing *D*-statistics using the qpDstats module of AdmixTools v7.0.2 [60] and visualized via R package ggplot2 v3.3.6 [54]. The population WBA was set as an outgroup and the events with |Z-score| > 3 were considered to be significant [61].

We also inferred 1–9 migration events via TreeMix v1.13 [59] among Yunnan indigenous pigs, WBA, and three European commercial pig breeds under two scenarios: (1) without root group, (2) setting Duroc pigs as the root group to construct a ML tree and test migration events and corresponding residuals by setting 1000 SNPs per window.

### Genome-wide scan for selection signature through XP-EHH method

To identify selective sweeps and potentially selected loci during domestication, we carried Cross Population Extended Haplotype Homozygosity (XP-EHH) method via XP-EHH software [62] by pairing each Yunnan indigenous breed (experimental group) with WBA (reference group) in the study. Before calculating XP-EHH scores, we inferred haplotypes with the parameters: -KL10 -KU30-Ki5 via *fastPHASE* software [63]. We treated the top 0.5% right-tail/ left-tail SNPs as significant SNPs detected in populations and visualized the results using R package CMplot v4.1.0 [64] and Venny v2.1.0 [65]. The following downstream analyses were mainly based on the potential SNPs detected in Yunnan indigenous pigs and WBA through XP-EHH method.

To further validate the XP-EHH results and highlight the significant adaptive evolutions during the domestication, we also employed the PCAdapt [66] method. This method utilizes principal component analysis to adjust for population structure and detect loci associated with adaptation with strength threshold, implemented through the R package pcadapt [67]. We selected K = 2 for the group pairs BS-WBA, DNXE-WBA, GLGS-WBA, and SB-WBA, and potential SNPs were identified by computing the False Discovery Rate (FDR, α = 0.05) of the *P*-values associated with Mahalanobis distance estimated by PCAdapt, using the R package *q*valu e[68].

### Functional annotation and QTL region permutation

To explore the biological function of the signatures detected, we conducted the gene annotation, enrichment analysis of candidate genes, Quantitative Trait Locus (QTL) analysis, and QTL region permutation analysis based on the potential selection regions which were the 200 kb window of ±100 kb region around significant SNPs.

#### Gene annotation and enrichment analysis

We annotated candidate genes according to physical position based on the *Sus Scrofa* 11.1 genome which was downloaded in ENSEMBL [69]. We defined genes located in the potential selection regions as candidate genes. Combining the Gene Ontology (GO) [70] database and the Kyoto Encyclopedia of Genes and Genomes (KEGG) [71] database, we annotated and categorized the functions of candidate genes using R package clusterProfiler v4.4.4 [72] and the Bioconductor annotation package *org.Ss.eg**.db* were imported for whole genome-wide annotation. Then we further explored interesting gene clusters and visualized functional annotation analyses by implementing GO: biology process (BP) terms and KEGG pathways using hypergeometric distribution tests through R package clusterProfiler v4.4.4 [72]. The significant threshold was set as a *P*-value < 0.05.

To further validate the candidate genes consistently identified in Yunnan indigenous pigs using the XP-EHH and PCAdapt methods, we performed enrichment analyses of GO biological process terms and KEGG pathway functions. These analyses were based on the 66 unique genes (Fig. S9d) and aimed to further confirm the vital potential selection of genes in Yunnan indigenous pigs detected by XP-EHH. The enrichments analyses were carried out using the DAVID server [73], applying a significance threshold of *q*-value = 0.05.

#### QTL analysis and QTL region permutation

We counted the QTLs overlapped with the potential selection regions and their reported frequency based on the pig QTL database in the Animal QTL database (release45) [74]. And then we further explored the significant class of traits by conducting QTL permutation test via R package regioneR v1.24.0 [75] and a total of 10,000 permutation tests were performed. To ensure the QTL permutation test reliability, the QTLs with unknown physical positions or with QTL regions more than 1 Mb and the trait type with a QTL number below 100 were removed. The QTL permutation results with *P*-value < 0.05 were considered to be significant and visualized via the R package ggplot2 v3.3.6 [54].

### Patterns of genotype and LD block of one 265.09 kb region on chromosome 5

To further explore the evolutionary pattern of one potential selection region (chromosome 5:33.67–33.93 Mb), we conducted LD block analysis and genotype pattern of the 265.09 kb region in which four shared candidate genes were annotated in WBA and Yunnan indigenous pigs. We employed BCFtools v1.90 [76] program to extract the potential region and VCFtools v0.0.13 [77] program to recode the genotype pattern with the option “--012”, and then visualized by using the R package pheatmap v1.0.12 [78]. We also conducted LD block analysis of the seven SNPs via the Haploview v4.2 [79] software.

### PheWAS, RNA expression, and associated phenotypes of shared candidate genes in other mammals

We conducted the phenome-wide association analysis (PheWAS) in humans, together with exploring associated phenotypes, biological functions, and RNA expression of seven shared candidate protein-coding genes in other mammals through extensive international bioinformatics database tools.

As orthologous genes have similar functions among mammals, we conducted PheWAS based on the human GWAS data in the GWASATLAS database [37, 38] to explore the associated complex traits in humans. Briefly, we explored the GWAS summary statistics of 558 traits from 181 human GWAS with a sample size > 5000, and a *P*-value < 0.05 was considered significant. Then we classified the complex traits into 12 trait domains based on the available knowledge of tissue biology [80]. We visualized the PheWAS results using the R package pheatmap v1.0.12 [78].

We employed the PigGTEx [39] portal and HPA v21.1 [44, 81] to explore the expression of candidate genes across tissues and organs in pigs and humans. We also analyzed the significantly associated phenotypes in mice via the IMPC database [41, 82], which provided phenotype data after standardized physiological tests that were identified on the systematical single gene ‘knock out’ mice.

## Results

### Phylogenetic relationships and population structure of Yunnan indigenous pigs

**Fig. 1:**
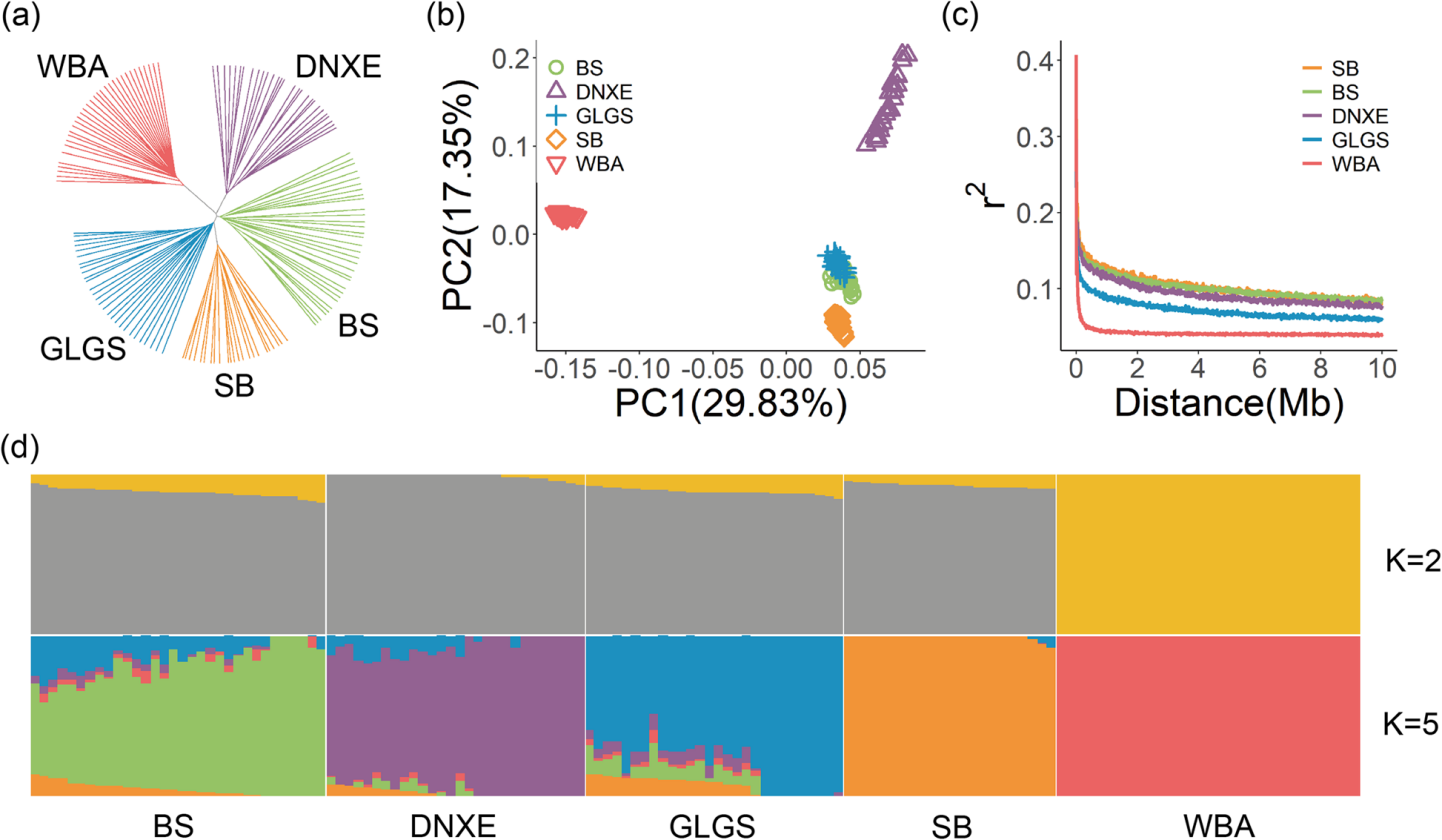
Population genetic structures of five pig breeds/populations. **a** Neighbor-joining phylogenetic tree of 144 pigs genotyped in Illumina Porcine SNP60K BeadChip. **b** Principal component analysis (PCA) result of 144 pigs on the first two PCs. **c** Linkage disequilibrium decay in the distance of 10 Mb. **d** Genetic ancestry compositions with the assumed number of ancestries *K* = 2 and *K* = 5, which had the lowest cross-validated error. BS, Baoshan pigs; DNXE, Diannanxiaoer pigs; GLGS, Gaoligongshan pigs; SB, Saba pigs; WBA, Asian wild boars

To clarify the population genetic structure among Yunnan indigenous pigs, we analyzed 144 individuals using autosomal SNPs (Fig. 1). In NJ tree analysis, all Yunnan indigenous pigs from the same breeds and WBA were found to cluster together (Fig. 1a). We also conducted PCA and structure analysis to further assess the population genetic relationship. The PCA results denoted that 29.83 and 17.35% of the genetic variance were attributed to the first two PCs, respectively. As PC1 separated the Yunnan indigenous pigs from WBA and PC2 divided Yunnan indigenous pigs into two clusters: DNXE and the other Yunnan pigs, five populations were clustered by breeds clearly (Fig. 1b). We estimated the proportions of Yunnan indigenous pigs and WBA lineages using ADMIXTURE using K from 2 to 6 (Fig. S2). The genomes of Yunnan indigenous pigs contained sectional proportions of WBA ancestry. It is appropriate to divide all individuals into five clusters with a minimum CV error of 0.51218 when *K* = 5. As an LD distance of 10 Mb was calculated (Fig. 1c), we observed higher LD levels in Yunnan indigenous pig breeds than WBA, which was commonly consistent with our perceptions that domestic pigs had lower genomic diversity than WBA population caused by artificial selection.

In the context of a broad panel of breeds, the population genetic analyses indicated that the classification of the samples from four Yunnan indigenous pig breeds, WBA, and three European commercial pig breeds was reasonable. Furthermore, the ancestry origin of Asian pigs and European pigs was found to be different (Fig. S3). The Asian pigs were separated from the European pigs through PC1, while PC2 divided the three European commercial pig breeds into two clusters: DUR, LWT, and LDR. PC3 divided Asian pigs into two clusters: WBA and Yunnan indigenous pigs (Fig. S3a). Phylogenetic networks analysis demonstrated that all samples were clustered by breeds clearly, with the exception of a few BS samples that were distributed in clusters separate from the main breed clusters, as seen in Fig. S3c. The genomes of Asian pigs (WBA and Yunnan indigenous pigs) differed from those of European commercial pigs (Duroc, Landrace, and Large White breeds) were different (Fig. S3b, *K* = 2). The genomes of the Yunnan indigenous pigs exhibit fractional proportions of WBA ancestry (Fig. S3b, *K* = 3) and BS pigs contained sectional proportions of Duroc ancestry (Fig. S3b, K = 4). It is appropriate to categorize all individuals into eight clusters clearly with the minimum CV error = 0.51445 when *K* = 8 (Fig. S3b, *K* = 8).

In the context of a broad panel with European commercial pigs, it is clear that Asian pigs and European pigs exhibited distinct genetic backgrounds (*K* = 2). Furthermore, Yunnan indigenous pigs originated from Asian wild boars (*K* = 3). Within the Yunnan indigenous pigs cluster, DNXE (*K* = 5), SB (*K* = 7), GLGS and BS (*K* = 8) were distinguished by breeds. In addition, BS and GLGS had relatively mixed genetic backgrounds of several breeds, and BS pigs contained more sectional proportions of Duroc than WBA ancestry (*K* = 8, Fig. S3b).

### Introgression analyses and genetic diversity of Yunnan indigenous pigs

**Fig. 2:**
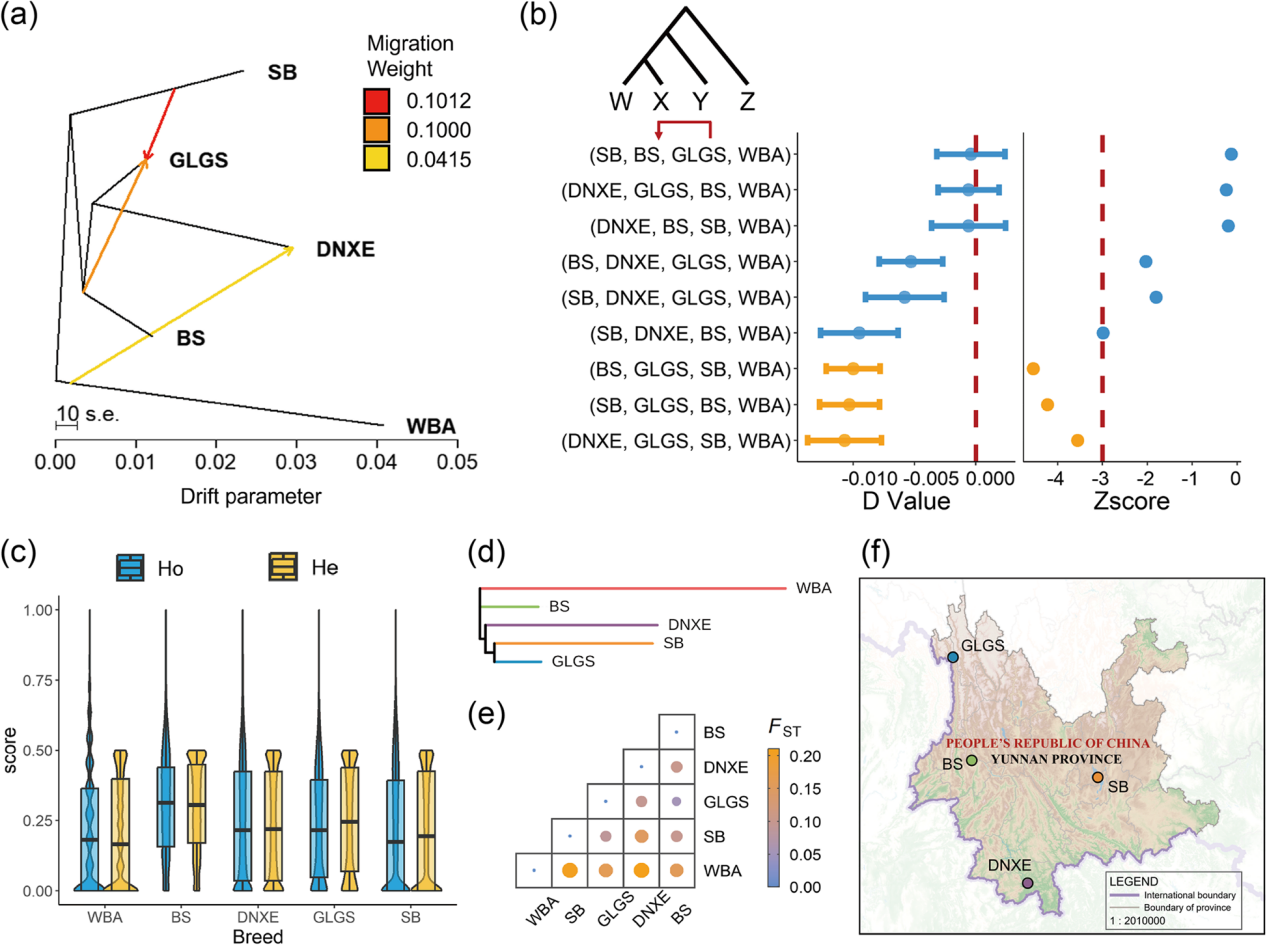
Migration analysis and genetic differentiation among the populations. **a** TreeMix introgression among the populations when m = 3. **b** *D*-statics test by AdmixTools among the populations. A negative *D* value indicated the introgression event was from group Y to X and the significant introgression events were plotted in yellow (Zscore < − 3). The left chart is *D* value and the right is Zscore. The error bars were *D* value ± SE. **c** Genome-wide observed heterozygosity (Ho) and expected heterozygosity (He) of four Yunnan pig populations and WBA samples. **d** *F*_ST_-based maximum likelihood tree of five populations. **e** Heatmap plot of genetic differentiation among five populations based on *F*_ST_. **f** Geographical distribution map of Yunnan pig populations. BS, Baoshan pigs; DNXE, Diannanxiaoer pigs; GLGS, Gaoligongshan pigs; SB, Saba pigs; WBA, Asian wild boars

We searched for potential migration events between Yunnan breed pigs and WBA using the approaches of Treemix and *D*-statistics. We performed the drift models assuming 1–5 gene flow events and accessed the residence in TreeMix analysis (Fig. S4). It revealed that three genomic introgression events happened from BS to GLGS, SB to GLGS, and WBA to DNXE (Fig. 2a). We also carried on *D*-statistics to further investigate the migration events among the Yunnan indigenous pigs. Three potential migration events were detected: (i) BS were closer to GLGS and (ii) SB was closer to GLGS than to BS or DNXE (Fig. 2b**,** Table S1), which were consistent with Treemix analysis results and commonly agreed with the geographical distribution (Fig. 2f). For the migrations among European commercial pigs, WBA and Yunnan indigenous pigs, the ML trees constructed under (1) unroot mode and (2) setting Duroc pigs as the root group showed that WBA and four Yunnan indigenous pig breeds were clustered in one clade (Fig. S5). It revealed that three genomic introgression events happened from DUR to BS, from LWT to DNXE, from SB to WBA, and from DNXE to BS (Fig. S5).

The average Ho (He) for WBA, BS, DNXE, GLGS, and SB were 0.205(0.198), 0.309(0.296), 0.243(0.234), 0.240(0.245) and 0.224(0.213). Based on the inferred population structure in Treemix, we found the BS population which was the most basal split within Yunnan indigenous pigs to have the highest heterozygosity, and the heterozygosity of the remaining populations with a progressive splitting showed a decrease in heterozygosity (Fig. 2c). The ML tree built by *F*_ST_ distances based on populations depicted clusters (Fig. 2d) that commonly agreed with their relationships derived from possible introgression and admixture events: (i) SB and GLGS were on the same branch; (ii) BS and DNXE were clustered together with SB and GLGS. While *F*_ST_ among Yunnan indigenous pigs was 0.053–0.102, *F*_ST_ was 0.158–0.210 between WBA and Yunnan indigenous pigs (Fig. 2e), which evidenced that there was a high (0.15 < *F*_ST_ < 0.25) genetic differentiation between Yunnan domestic pigs and WBA.

### Signature of artificial selection detected in Yunnan indigenous pigs and WBA

**Fig. 3:**
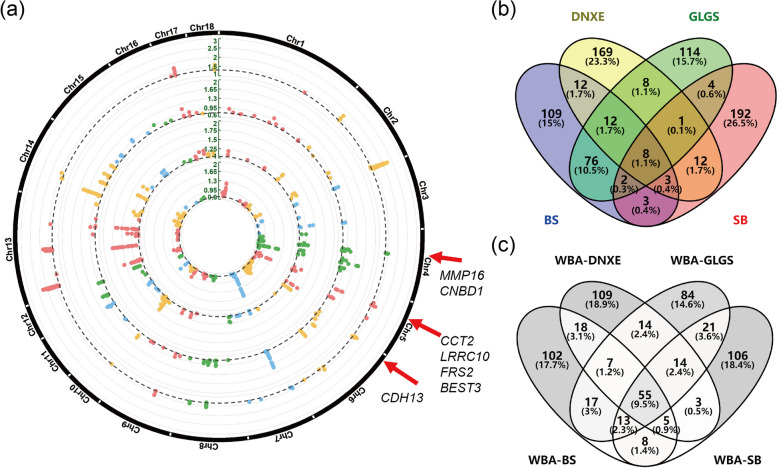
Genome-wide distribution of selection signatures identified in Yunnan indigenous pigs via XP-EHH method. **a** Circle Manhattan plot of significant SNPs on the whole genome. The rings from inner to outsider showed results of BS, DNXE, GLGS, and SB. The black dashed lines represent a 99.5% threshold value. The genes were breed-sharing in Yunnan indigenous pigs. **b-c** Venn diagram about the candidate SNPs detected in Yunnan indigenous pigs and WBA samples. BS, Baoshan pigs; DNXE, Diannanxiaoer pigs; GLGS, Gaoligongshan pigs; SB, Saba pigs; WBA, Asian wild boars

We proceeded the top 1% outliers (i.e., 450 SNPs) as significant SNPs in every pair XP-EHH analysis (Fig. 3, Fig. S6 and S7, Table S2). The raw XP-EHH scores of significant SNPs in BS, DNXE, GLGS, and SB were greater than 0.7063, 1.0494, 0.7594, and 1.3175, and in WBA were lower than − 1.1075, − 0.9529, − 1.0558 and − 0.7471, respectively. We found many breed-sharing and breed-specific significant SNPs in different breeds. There were peaks on *Sus Scrofa* chromosome 1 (SSC1), SSC3, and SSC15 for WBA pigs and 55 shared SNPs were mainly on SSC1:84.21–86.24 Mb (Fig. S7). Four Yunnan indigenous pigs shared 6 SNPs on SSC4: 48.93–49.5 Mb and 2 SNPs on SSC5: 33.88–33.90 Mb (Fig. 3a). The breeds-specific SNPs showed a heterogeneous distribution on the whole genome, and selection signatures for SB were detected on SSC2 and SSC13. What’s more, 98 shared SNPs were detected for GLGS and BS, with one clear peak on SSC7 (Fig. 3a and b).

### Gene annotation, QTL, and QTL permutation analysis for candidate SNPs

We annotated 225, 156, 213, 140, and 493 genes with official gene names on BS, DNXE, GLGS, SB, and WBA (Fig. S8, Table S3). Seven protein-coding genes: matrix metallopeptidase 16 (*MMP16*), cyclic nucleotide-binding domain containing 1 (*CNBD1*), fibroblast growth factor receptor substrate 2 (*FRS2*), leucine-rich repeat containing 10 (*LRRC10*), chaperonin containing TCP1 subunit 2 (*CCT2*), bestrophin 3 (*BEST3*) and Cadherin 13 (*CDH13*) and one Non-coding RNA gene *U6* were commonly detected in four Yunnan indigenous pig breeds (Fig. 3). We further explored biological functions of the protein-coding genes in the following study. Apart from *FRS2*, *CCT2*, *LRRC10,* and *BEST3* detected on the shared region on SSC5, gene *ENSSSCG00000000500* (*RAB3IP*) was also annotated on the region and it was validated to be involved in the phenotypes of increased mean corpuscular volume and increased mean corpuscular hemoglobin [41] and the diseases caused by decreased bone mineral density in mice [83].

In the selection signature analysis conducted using the PCAdapt method, we identified a total of 287, 794, 588, and 669 significant SNPs in the BS-WBA, DNXE-WBA, GLGS-WBA, and SB-WBA groups, separately. Moreover, we annotated 452, 1117, 911, and 1033 genes with official names in each respective group (Fig. S9, Table S4). Among these, 307 SNPs and 632 genes were consistently detected in at least 2 groups, indicating a certain level of similarity in the selection patterns observed in Yunnan indigenous pigs.

Among the candidate genes identified through XP-EHH and PCAdapt, 30 (13.33%), 15 (9.62%), 27 (12.68%), and 24 (17.14%) genes were consistently detected in BS, DNXE, GLGS, and SB pigs, respectively (Fig. S9c). Subsequently, we compiled the overlapping genes found in Yunnan indigenous pigs and performed enrichment analyses on the 66 distinct genes to explore the biological functions of potentially selected genes in Yunnan indigenous pigs (Fig. S9d).

#### QTL analysis and QTL region permutation

**Fig. 4:**
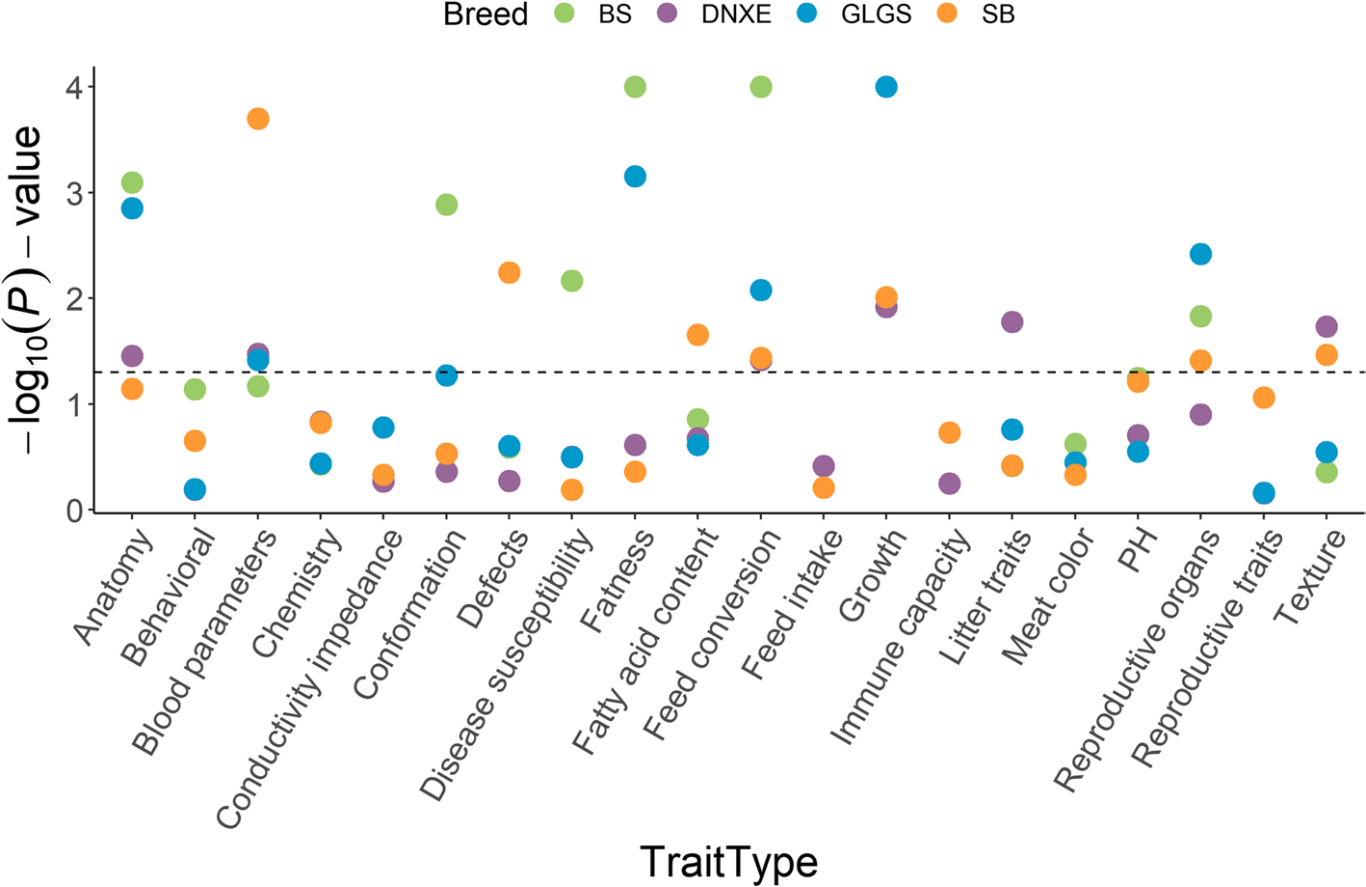
The QTL permutation of the potential selection regions in Yunnan indigenous pig. The *y*-axis represents the -log_10_ *P*-value of the QTL annotation trait type and the x-axis represents the trait type, which is the trait that the QTL was annotated significantly. The color of the point encodes different breeds. BS, Baoshan pigs; DNXE, Diannanxiaoer pigs; GLGS, Gaoligongshan pigs; SB, Saba pigs; WBA, Asian wild boars

While QTLs overlapped with potential SNP regions in WBA were almost annotated on the ‘Meat and Carcass’ class, such as Drip loss QTL annotated on the linked significant SNPs peak on SSC1:84.02–86.51 Mb, in Yunnan pigs, the QTL-related traits with significant *P*-value (*P*-value < 0.05) were in ‘Production’ class of ‘growth’ and ‘feed conversion’ types, ‘Reproduction’ class of ‘litter traits’ and ‘reproductive organs’, together with ‘Health’ class of ‘disease susceptibility’ and ‘blood parameters’ types (Fig. 4, Table S5). Several breed-specific signatures with high raw XP-EHH scores associated with breed germplasm characteristics were also detected in Yunnan indigenous pigs (Table S3). The top 10 SNP with higher XP-EHH scores in SB pigs were most overlapped with the *actinobacillus pleuropneumoniae* susceptibility QTL and significant SNPs on SSC13 in DNXE were most overlapped with the Interleukin-12 level QTL. Fat androstenone level QTL were most reported on SSC14:38.50–39.21 Mb containing 17 adjacent SNPs in GLGS pigs. The cannon bone circumference QTL reported only in BS and GLGS pigs overlapped with several shared and continuous SNP on the region of SSC7.

In addition, we conducted gene annotation and QTL analysis based on breed-sharing and breed-unique SNPs to further investigate the sharing selection signatures and germplasm resources on Yunnan indigenous pigs (Table S6). The genes annotated on the 8 shared SNPs were *MMP16*, *CNBD1*, *LRRC10*, *FRS2*, *CCT2,* and *BEST3*, which were nearly common with the 8 shared genes.

### Enrichment analysis of candidate genes in WBA and Yunnan indigenous pigs

While the candidate genes of WBA were enriched in several life-fundamental and life-necessary KEGG pathways and GO terms associated with cellular metabolism, organism development, and growth (Fig. S10e), Yunnan pigs were more enriched in those that matched the changes in immune performance and nervous development system (Fig. S10, Table S7), in addition to being enriched in basal metabolic and development function. We identified several genes (e.g., *ITPR2*, *ITPR3*, *ATP2B1*, *KCNK2*, etc.) enriched on the functions related to taste transduction, salivary secretion, and gastric acid secretion. The genes (e.g., *SLC1A1*, *ATP2B1*, *SMYD3*, *VIM*, *STAT4*, etc.) were enriched on the term of (cellular) response to oxygen-containing compound. Genes enriched on an abundant of pathways associated with the regulation, activation, and proliferation of lymphocyte, cytokine and leukocyte, which underlined the stronger immune performance in Yunnan pigs, such as the GO: BP terms which shared candidate genes in BS and GLGS enriched on were clustered in the immune system biofunctions (Fig. S10f).

For enrichments analysis of 66 overlapped candidate genes in Yunnan indigenous pigs through XP-EHH and PCAdapt, genes *CAMK2A*, *ITPR3*, *PLCB1*, and *CAMK2G* were jointly enriched significantly on the KEGG pathways ssc04720: Long-term potentiation (*q*-value = 0.03015), ssc04971: Gastric acid secretion (*q*-value = 0.03297), ssc04911: Insulin secretion (*q*-value = 0.03572), ssc04750: Inflammatory mediator regulation of TRP channels (*q*-value = 0.03987), and ssc04725: Cholinergic synapse (*q*-value = 0.04011), which played an important role in the domestication of pigs (Table S8).

### PheWAS in human and related trait domains of seven shared candidate genes

**Fig. 5:**
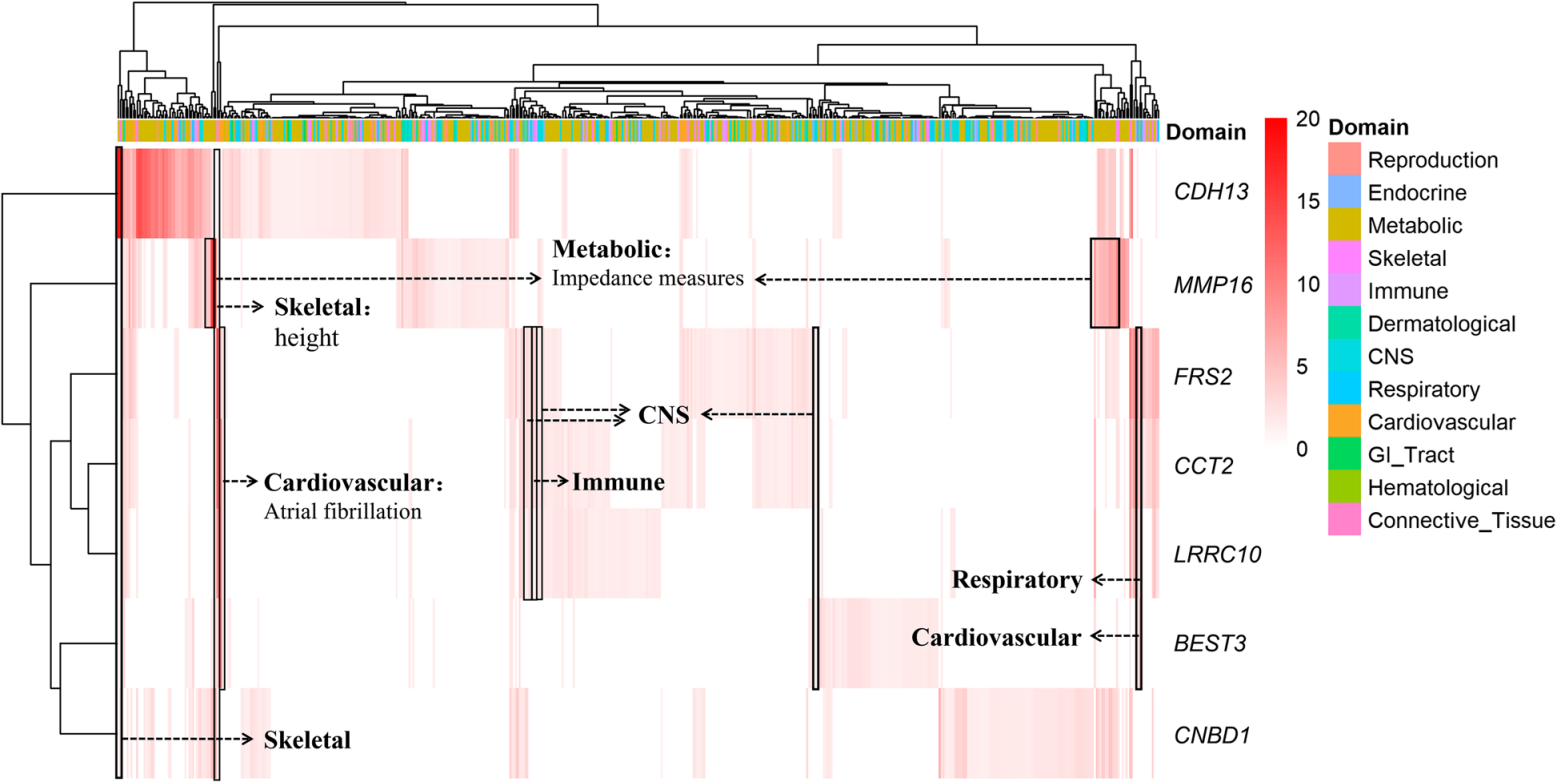
Phenome-wide association study heatmap in humans of 7 candidate protein-coding genes. The GWAS summary statistics of 558 traits from 181 human GWAS with sample size > 5000 and *P*-value < 0.05 were considered and the traits were classified into 12 trait domains. Color corresponds to the -log_10_*P*-value. The *y*-axis represents the genes, and the *x*-axis represents the traits, which are colored according to the trait domains. CNS, central nervous system; GI_tract, gastrointestinal tract

The phenotypes of complex traits significantly associated with genes explain to some extent the biological functions of many protein-coding genes. As we identified shared orthologous genes with high confidence between pigs and humans, we conducted the PheWAS on humans based on candidate protein-coding genes (Fig. 5, Table S9). The genes *FRS2*, *CCT2*, *LRRC10,* and *BEST* were annotated on two shared candidate SNPs on SSC5. They were found to be specifically expressed specifically in the domains of cardiovascular, central nervous system, respiratory, and immune. *MMP16* was significantly and specifically associated with impedance measures (metabolic domains) in humans, which is correlated to the phenotype of chronic pain resistance. The genes *MMP16* (heel bone mineral density), *CDH13* (adiponectin level), and *FRS2* (height) displayed noteworthy connections with traits associated with metabolism and skeletal tissues in humans.

### Region’s patterns of LD block and haplotype in WBA and Yunnan indigenous pigs

**Fig. 6:**
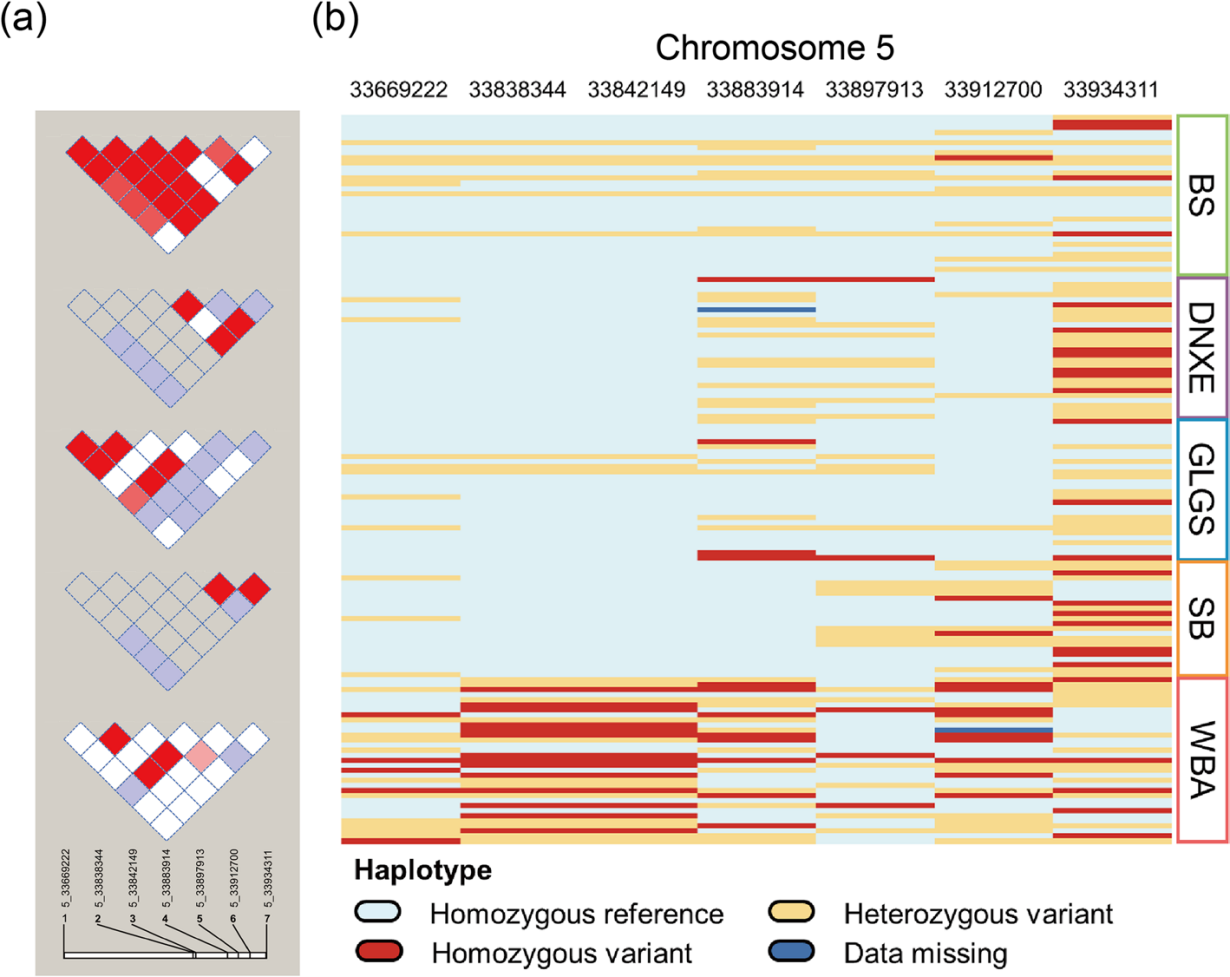
Haplotype and LD block patterns of region Chr5: 33669222–33,934,311 for Yunnan indigenous pigs and WBA samples. **a** Linkage disequilibrium (LD) block pattern in the region of BS, DNXE, GLGS, SB, and WBA. LD blocks are marked with triangle, LD value (D′) between SNP pair are detailed in boxes (D′ = 0–1) and D′ = 1 indicates perfect disequilibrium. **b** Haplotype patterns of the region. Each row represents an individual and its breed name is marked in the right colored box. Each column represents one SNP in the region. BS, Baoshan pigs; DNXE, Diannanxiaoer pigs; GLGS, Gaoligongshan pigs; SB, Saba pigs; WBA, Asian wild boars

Based on the former studies, we detected that genes *FRS2*, *CCT2*, *LRRC10,* and *BEST3* were annotated on the same region (Chr5: 33.67–33.93 Mb) and the results of PheWAS showed they were associated significantly (*P*-value < 0.05) with similar trait domains. To further investigate the interesting 265.09 kb region, we extracted the region and conducted the LD block and haplotype analyses in Yunnan indigenous pigs and WBA samples (Fig. 6). Three homozygous SNPs (i.e., without MAF value) in DNXE and SB were plotted as blank blocks. Consistent with haplotype pattern analysis, Yunnan pigs shared a similar LD block pattern in the genome region, which differed from the WBA population.

### Biological functions and RNA expression across tissues of shared genes

**Fig. 7:**
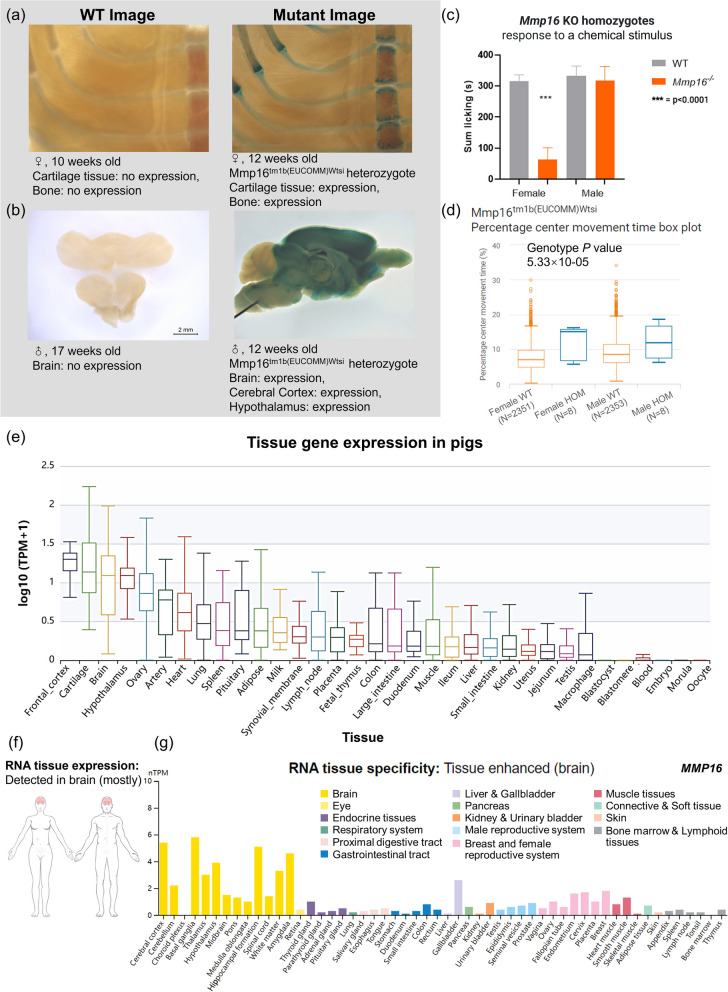
Gene expression and biological phenotypes regulated by *MMP16* in mammals. **a-b** Specific *LacZ* expression for adult *Mmp16*^+/−^ mutant mice and wild type (WT) mice on tissue brain and bone in the stage of adult. **c** Increased pain resistance assessment on *Mmp16*^*−/−*^ KO mice and WT mice. **d** The decreased anxiety-related response on *Mmp16*^−/−^ mice and WT mice. **e** RNA expression overview on pigs. **f-g** RNA expression atlas anatomogram and RNA expression overview across tissues. nTPM, normalized expression levels. Color coding is based on tissue groups, each consisting of tissues with functional features in common. Image credit: **a**-**d**, International Mouse Phenotyping Consortium [41], www.mousephenotype.org. **e**, PigGTEx-Portal [39], http://piggtex.farmgtex.org/. **f**-**g**, Human Protein Atlas [44], www.proteinatlas.org. Images are available at the following URL: v21.proteinatlas.org/ENSG00000156103-MMP16/tissue

*MMP16* expressed specifically in bone, cartilage tissue (Fig. 7a), and the tissues of the brain, cerebral cortex, and hypothalamus (Fig. 7b) in the life stage of adult through bacterial beta-galactosidase (*lacZ*) activity assessment of WT mouse and mutant mouse (*Mmp16*^tm1b(EUCOMM)Wtsi^ heterozygote). *Mmp16* KO homozygotes female mice showed a response to the pain of chemical stimulus (*P*-value < 0.0001, Fig. 7c) by measuring the time spent licking in the condition of formalin injection**.** Figure 7d showed *MMP16* was significantly associated with phenotype “decreased anxiety-related response” through measuring Percentage center movement time in 8 females and 8 male mutants compared to 2372 female and 2373 male controls in mice. The mutants are for the Mmp16^tm1b(EUCOMM)Wtsi^ allele. In the PigGTEx Atlas project, *MMP16* was generally expressed in vivo and highly expressed in the brain (frontal cortex, hypothalamus) and cartilage, ovary, artery, and heart tissues in pigs (Fig. 7e). RNA expression of *MMP16* generated in HPA had specificity in the brain tissues in human (Fig. 7f-g) and was clustered in brian-neuronal signaling. Likely, associated phenotypes and expression of RNA and protein of *FRS2*, *CCT2*, *BEST3,* and *LRRC10* showed them were consistently correlated to disease pathogenesis and vascular/cardiac function according to HPA and IMPC (Fig. S11).

## Discussion

### Phylogenetic relationship and genetic diversity of Yunnan indigenous pigs

In our study, we analyzed the genetic diversity and migration events, adaptive evolution under artificial selection based on the obtained SNP60K BeadChip genotypes of 144 privately sequenced pigs consisting of Asian wild boars and four breeds of Yunnan indigenous pigs with excellent traits such as good meat quality, disease resistance and production performance. The sample sizes of the populations in the current study were comparable to those in similar studies [84–86] and were suitable for analyses of population structure, genetic diversity, introgression, and selection signatures. Analyses performed with K = 2 and K = 5 showed that the origin of Yunnan indigenous pigs was less than 0.12, suggesting that there were almost no recent hybrids between WBA and domesticated pigs in the study. When K = 2, four Yunnan indigenous pig breeds were separated from WBA, the populations of BS, GLGS, SB, and DNXE had 11.5495, 10.9667, 7.1858, and 1.2112% genetic material of WBA populations, respectively. And when K = 5, the indices were 2.1881, 1.8606, 0.001, and 0.8146%, respectively. As BS and GLGS pigs were distributed in the southern zone of Yunnan, the low *F*_ST_ index between them (Fig. 2e) showed that they had slightly weak genetic differentiation and relatively high genetic similarity compared with other Yunnan breeds, which was consistent with previous studie s[87]. However, we also found that the migration analyses (Fig. 2a-b) suggested that there may have been a strong migration event may have occurred from SB to GLGS pigs, which still needs further investigation.

In addition, population genetic analysis in this study showed that DNXE pigs have a relatively independent (*K* = 3, Fig. S2) and pure genetic background compared to other Yunnan endemic pigs. The main breeding area of DNXE pigs is located in Xishuangbanna Dai Autonomous Prefecture, which is located in the southern part of Yunnan at a low altitude and borders with Southeast Asian countries. Given its unique genetic background and geographical location of biodiversity importance, we speculate that a phylogenetic analysis among DNXE pigs, domesticated pigs and wild boars in Southeast Asia may be interesting and would help us to further understand the domestication and migration events between Asian domestic pigs and Southeast Asian wild boars.

Among the genetic heterozygosity analysis indices, He is suitable for measuring population genetic diversity, with higher values indicating greater genetic diversity and lower genetic concordance in the population [88]. In this analysis, the highest average He was found in BS, GLGS’s was greater than DNXE’s, and the lowest average He was found in SB (Fig. 2c). Quan et al. [89] employed mitochondrial DNA D-loop in native Yunnan pigs and showed that the highest value of haplotype diversity was found in BS pigs and the lowest in GLGS pigs, the highest value of nucleotide diversity was found in DNXE and the lowest was also found in GLGS pigs. Using microsatellite DNA markers, Ouyang et al. [87] found that GLGS pigs had higher level average He level than BS pigs, and the genetic diversity of DNXE was lower than that of other Yunnan indigenous pigs which also demonstrated the breed need to strengthen the conservation strategies for DNXE pigs. The slightly different results of the analysis may be due to differences in the sampled individuals, genetic markers, and detection methods. For the lower genetic diversity of DNXE pigs compared to some other pig breeds, a previous study found out that the ancient (up to 50 generations ago) inbreeding had a greater impact than recent (within the last five generations) inbreeding within DSE pigs by calculating the inbreeding coefficients based on runs of homozygosity [90].

### Breed sharing and unique signatures of artificial selection in Yunnan pigs

In this study, we found that several genes such as *ITPR2* and *ITPR3* were detected in Yunnan indigenous pigs to be repeatedly enriched on several functional pathways such as taste transduction [91], Calcium signaling pathway [92], salivary secretion (Table S7), which may contribute to the adaptation of pigs to a stable feed supply from humans or supplemental feeding compared to WBA that have to endure starvation. With the multiple evidence from IMPC, human PheWAS results and HPA (Fig. 5, Fig. S11, Table S9), *FRS2*, *CCT2*, *LRRC10,* and *BEST3* were correlated with adaptive evolution in the metabolic and respiratory domains, which were consistent with previous research [93–95].

Notably, we detected 98 shared significant SNPs and 87 candidate genes annotated in BS and GLGS pigs living in adjacent residences and our analyses supported that gene introgression may have occurred from BS to GLGS pigs. These breed-sharing signatures were more enriched in the biological process terms of immune cell activation and regulation of immune cells and immune response process (Fig. S10, Table S7), and the overlapping QTLs were more associated with reproductive organs (Table S7). In addition, two human-mediated genes *PACSIN1* and *GRM4,* which had been reported to be associated with taste transduction and feed intake and body weight in European commercial pigs [96] (Yorkshire) and Chinese indigenous pigs [90, 97] (Meishan and Diannanxiaoer pigs), were also found to be associated with feed adaptation and body growth in the process of domestication in our study. As BS pigs have a large body size and GLGS pigs are small mountain pigs, how *PACSIN1* regulates the body size in indigenous pigs still needs to be elucidated by more level data analysis, such as whole genome sequence data and transcriptome expression data.

Several breed-specific selection signatures were also detected in our study. Several adjacent potential SNPs were detected on SSC2:148.78–151.43 Mb (68 SNPs) and SSC13:14.46–15.92 Mb (19 SNPs), SSC13:16.51–17.35 Mb (25 SNPs) in SB pigs were detected to be significantly associated with humoral innate immune defence and susceptibility to porcine salmonellosis in the spleen. These signatures would provide information on disease resistance and immune competence that could be used in selective breeding and further research to improve therapeutic and prophylactic measures. In addition, the QTLs overlapped with SSC14:110.68–113.99 Mb which was detected in the XP-EHH method for DNXE pigs and WBA were correlated to lipid deposition and abundant fatty acid content such as saturated fatty acid and stearic acid for excellent pork flavor.

### Leveraging bioinformatics database tools to identify the distinct roles of *MMP16* in complex traits in mammals

In our study, we found that *MMP16*, an artificially selected gene in domestic pigs, is significantly associated with bone and brain development. *MMP16* has been shown to be significantly associated with pain symptoms and sensitivity in humans and mice [98, 99]. *MMP16* has been reported to be a critical regulator of extracellular matrix degradation in complex physiological processes, such as cartilage formation and regeneration [100, 101] and neurological development disorders [102–104]. The gene has previously been shown to enhance axonal and synaptic plasticity [105]. To our knowledge, this is the first study to demonstrate the association between *MMP16* and its adaptive domestication in Chinese indigenous pigs, and we supposed it may relate to the pain tolerance to metabolic diseases in pigs brought in artificial breeding. Our results suggest that the suppression of *MMP16* would directly lead to inactivity and insensitivity of neuronal activity and skeletal development in Chinese indigenous pigs. However, the exact role of *MMP16* in the brain development and cartilage generation of pigs during the domestication process needs to be discovered.

Nowadays, we still know relatively little aboutthe function of most genes in the pig genome, which is clearly illustrated by the results of selection signature analyses and genome-wide association studies. The significant loci, which were found to be associated with important traits, are usually still the starting point of more detailed studies that investigating the function of genes in the vicinity of the identified locus. To get closer to uncovering the multiple, pleiotropic functions of genes, making good use of the emerging extensive bioinformatics database tools will enable us to unravel the secrets of genetic codes faster and develop a further perception of the occurrence of domestication and the process of directional selection on demand, and even to adapt biological pigs that meet human medical needs in the near future.

## Conclusion

In summary, we explored the genetic characteristics and genetic diversity of four Yunnan pig breeds and the migration events among them. We also detected signatures of long-term artificial selection using XP-EHH and PCAdapt methods and leveraged multiple bioinformatics database tools to resolve the biological functions of the selected variants. The selection signatures in Yunnan indigenous pigs were found to correlate with body growth, immune, and reproductive performance, as well as a cardiovascular-associated and immune-associated genome region on chromosome 5. We also found an artificially selected gene, *MMP16,* associated with metabolism and brain development, and several genes involved in dietary adaptation, including *PASCIN1*, *GRM4*, *ITPR2,* etc. Our findings may help to improve the understanding of variants caused by artificial selection in domesticated pigs and provide insight into the utilization of extensive bioinformatics database tools to decipher the pleiotropic functions of genes.

### Supplementary Information

**Additional file 1.** Figures S1-S11 and Tables S1-S9.

## Data Availability

All related data produced or analyzed in this study are available from the corresponding authors upon reasonable request.

## Abbreviations

BS: Baoshan pigs
DNXE: Diannanxiaoer pigs
GLGS: Gaoligongshan pigs
SB: Saba pigs
WBA: Asian wild boars
QC: quality control
PCA: Principal component analysis
LD: Linkage disequilibrium
IBS: Identity by state
XP-EHH: Cross population extended haplotype homozygosity
QTL: Quantitative Trait Locus
GO: Gene ontology
KEGG: Kyoto Encyclopedia of Genes and Genomes
IMPC: International Mouse Phenotyping Consortium
HPA: Human Protein Atlas
PheWAS: Phenome-wide association analysis
SSC: *Sus Scrofa* Chromosome
