# Supplementary material for: Exploring the mechanism of artificial selection signature in Chinese indigenous pigs by leveraging multiple bioinformatics database tools
Source: BMC Genomics. 2023 Dec 5;24:743. doi: 10.1186/s12864-023-09848-7 (PMC10699062; doi:10.1186/s12864-023-09848-7)
Supplement: Supplementary file 1 — Additional file 1. Figures S1-S11 and Tables S1-S9. [file 12864_2023_9848_MOESM1_ESM.zip › 02_Supplementary files/Additional file 10_Figure S8_Summary of candidate genes annotated in Yunnan indigenous pigs.pdf]

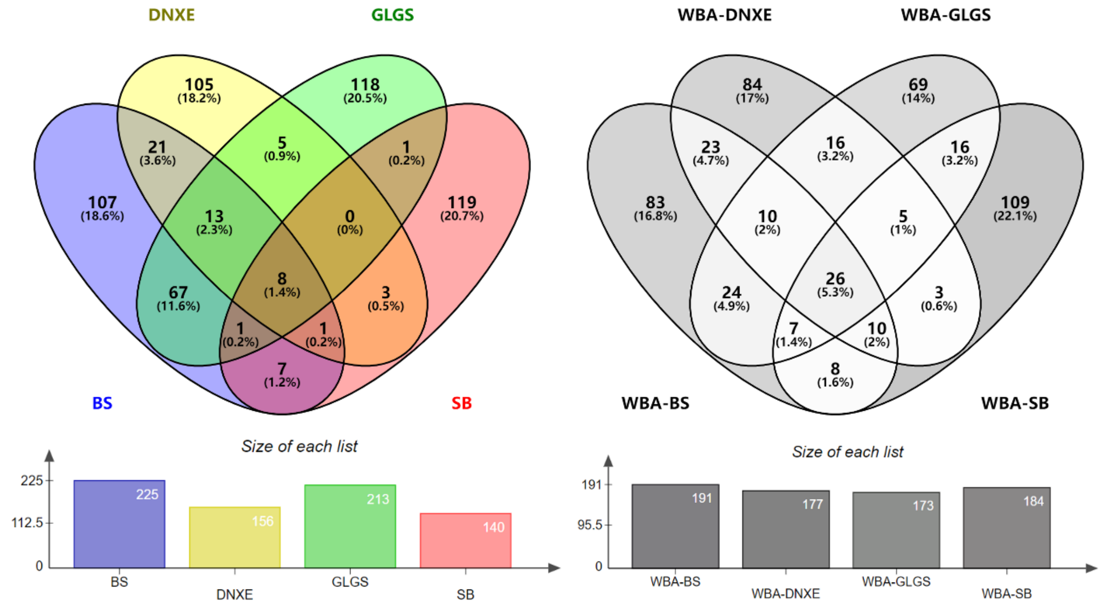

Figure S8 Summary of candidate genes annotated in Yunnan indigenous pigs and WBA samples. Venn diagram (top) and the size (bottom) of the candidate genes which were annotated in the candidate regions which were detected by XP-EHH analysis between various Yunnan pig breeds and WBA. The left panel showed the distribution of shared and unique selected genes (top) and the size of candidate genes (bottom) in the Yunnan domestic pig breed, while the right panel shows the candidate genes in WBA population in different analysis.
