# Supplementary material for: Exploring the mechanism of artificial selection signature in Chinese indigenous pigs by leveraging multiple bioinformatics database tools
Source: BMC Genomics. 2023 Dec 5;24:743. doi: 10.1186/s12864-023-09848-7 (PMC10699062; doi:10.1186/s12864-023-09848-7)
Supplement: Supplementary file 1 — Additional file 1. Figures S1-S11 and Tables S1-S9. [file 12864_2023_9848_MOESM1_ESM.zip › 02_Supplementary files/Additional file 12_Figure S9_The potential SNPs and candidate genes detected by PCAdapt method.pdf]

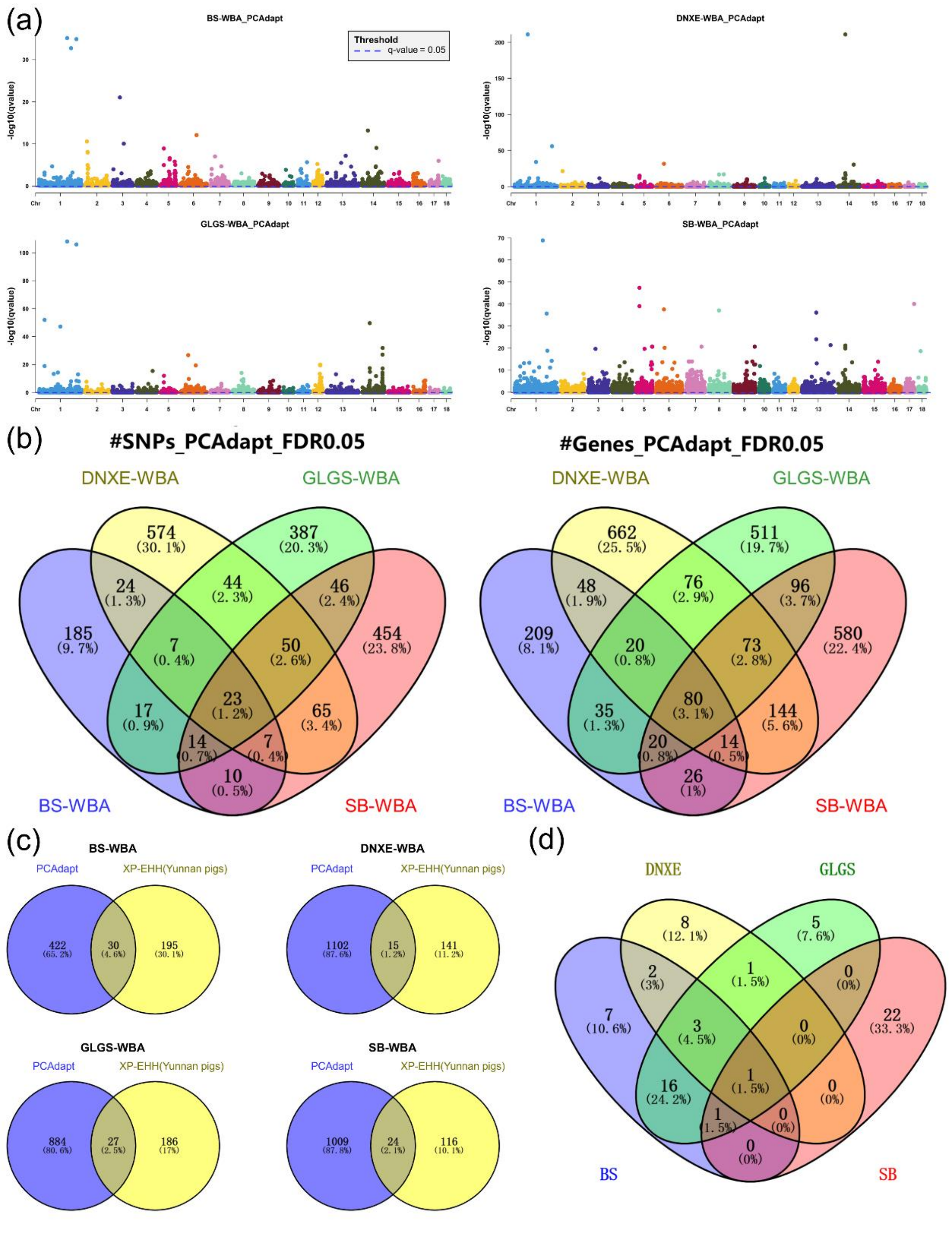

Figure S9 The potential SNPs and candidate genes detected by PCAdapt method. **a** Manhattan plot of potentially selective signatures detected in Yunnan pigs and WBA. The *x*-axis and *y*-axis represent chromosome number and the -log10(*q*-value). The dashed line in blue is corresponded to the threshold of *q*-value=0.05. **b** Venn diagram of potentially selected SNPs and candidate genes detected in four group pairs Yunnan indigenous pigs-Asian wild boars. **c** Venn diagram of candidate genes detected in four group pairs via PCAdapt and XP-EHH (only in Yunnan pigs). **b** The 66 unique candidate genes in Yunnan indigenous pigs. BS, Baoshan pigs; DNXE, Diannanxiaoer pigs; GLGS, Gaoligongshan pigs; SB, Saba pigs; WBA, Asian wild boars.
