# Supplementary material for: Exploring the mechanism of artificial selection signature in Chinese indigenous pigs by leveraging multiple bioinformatics database tools
Source: BMC Genomics. 2023 Dec 5;24:743. doi: 10.1186/s12864-023-09848-7 (PMC10699062; doi:10.1186/s12864-023-09848-7)
Supplement: Supplementary file 1 — Additional file 1. Figures S1-S11 and Tables S1-S9. [file 12864_2023_9848_MOESM1_ESM.zip › 02_Supplementary files/Additional file 16_Figure S10_GO and KEGG enrichment of candidate genes for Yunnan pigs and WBA.pdf]

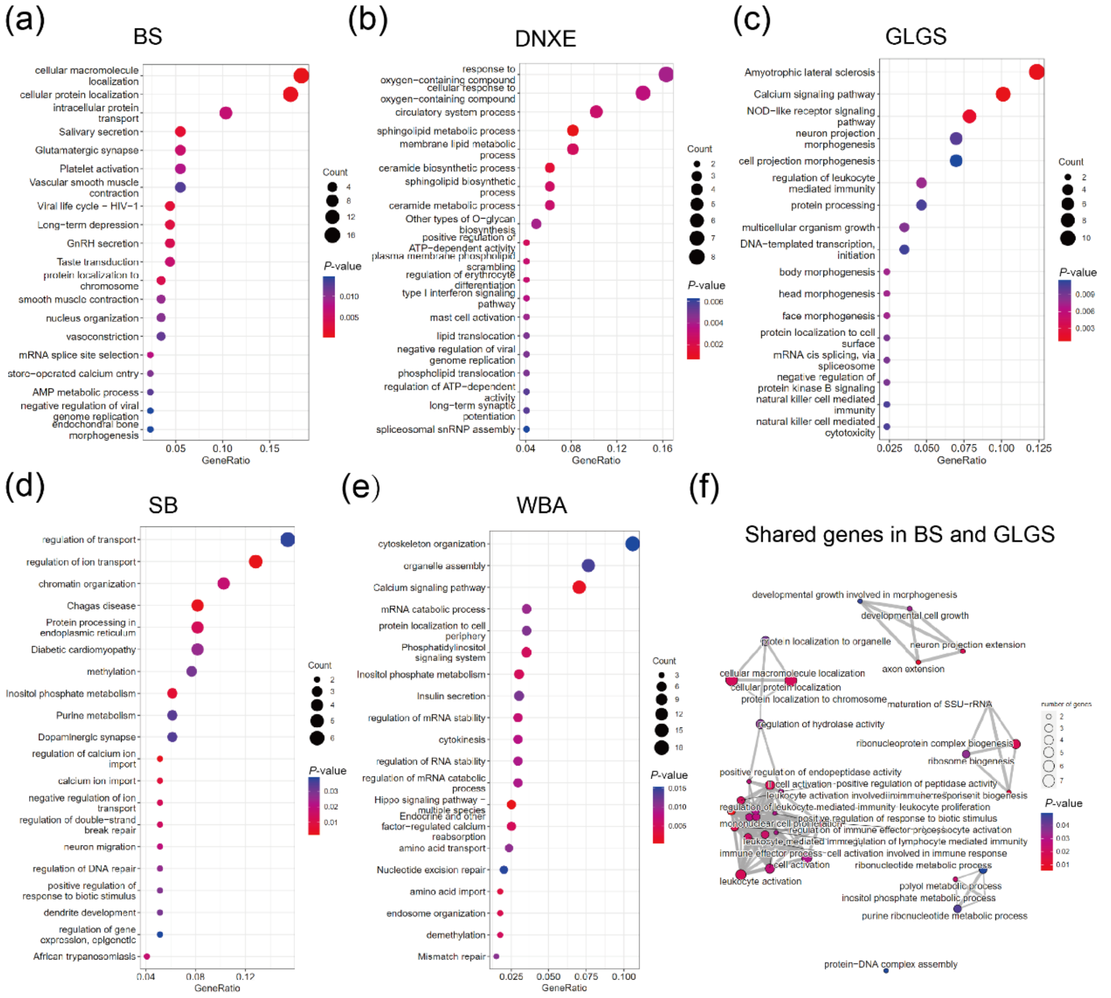

Figure S10 GO and KEGG enrichment of candidate genes for Yunnan pigs and WBA samples. **a-e** Bubble chart of the top 20 significant KEGG pathways and GO: biology process terms for BS, DNXE, GLGS, SB and WBA populations. **f** Summarized GO cluster chart of the GO: BP terms overrepresentation analysis for the shared genes in BS and GLGS pigs. In the bubble chart and bar chart, the *y*-axis represents the terms or pathways, and the *x*-axis represents gene ratio, which is the proportion of genes that enriched on the target term/ pathway in the candidate genes. The size of dot corresponds to the number of genes that enriched on the term/pathway and the color encodes the *P*-value as the legend showed.
