# Supplementary material for: Exploring the mechanism of artificial selection signature in Chinese indigenous pigs by leveraging multiple bioinformatics database tools
Source: BMC Genomics. 2023 Dec 5;24:743. doi: 10.1186/s12864-023-09848-7 (PMC10699062; doi:10.1186/s12864-023-09848-7)
Supplement: Supplementary file 1 — Additional file 1. Figures S1-S11 and Tables S1-S9. [file 12864_2023_9848_MOESM1_ESM.zip › 02_Supplementary files/Additional file 1_Figure S1_PCA results of new-added European commercial pigs.pdf]

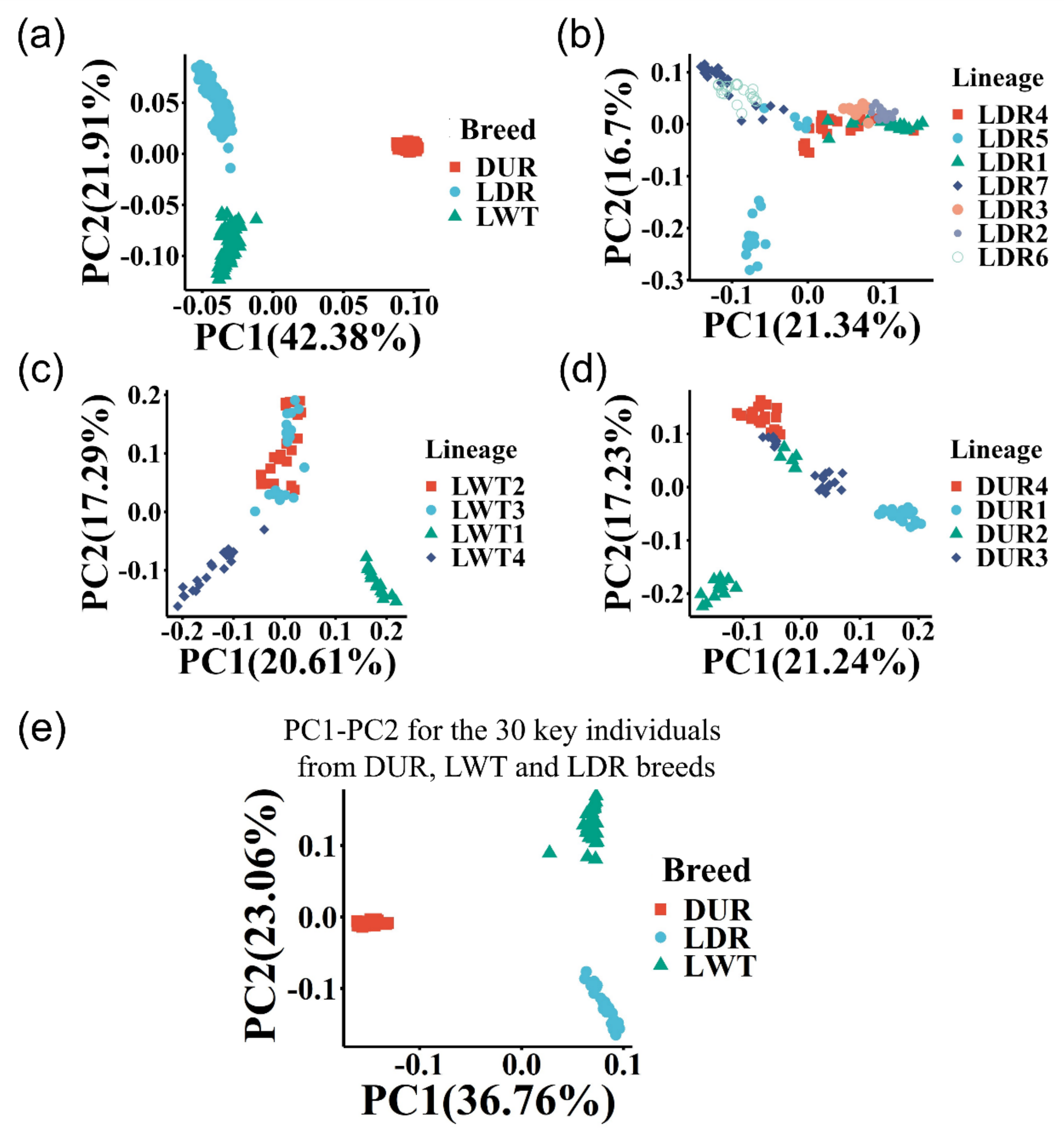

Figure S1 Principal component analysis overview of original and key individuals of new-added European commercial pigs. **a** PCA result of the original combined population which consists of 76 LWT, 130 LDR and 79 DUR. **b-d** PCA results of different lineages of three breeds. **e** PCA result of the combined population of 30 key individuals per breed. DUR, Duroc pigs; LDR, Landrace pigs; LWT, Large White pigs.
