# Supplementary material for: Exploring the mechanism of artificial selection signature in Chinese indigenous pigs by leveraging multiple bioinformatics database tools
Source: BMC Genomics. 2023 Dec 5;24:743. doi: 10.1186/s12864-023-09848-7 (PMC10699062; doi:10.1186/s12864-023-09848-7)
Supplement: Supplementary file 1 — Additional file 1. Figures S1-S11 and Tables S1-S9. [file 12864_2023_9848_MOESM1_ESM.zip › 02_Supplementary files/Additional file 2_Figure S2_Genetic population structure of Yunnan indigenous pigs.pdf]

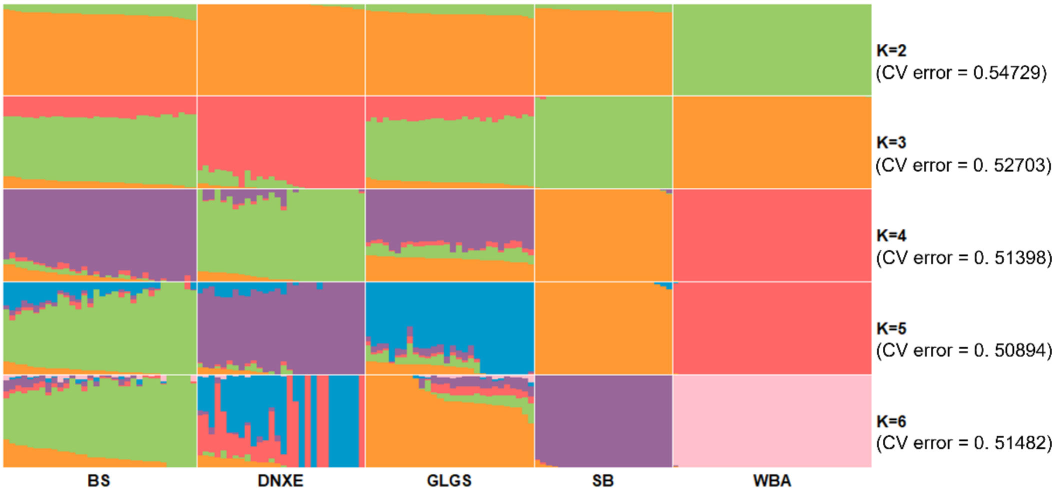

Figure S2 Genetic population structure of five pig populations from K=2 to K=6 by Admixture. The length of each colored segment represents the proportion of genome inferred from ancestral populations (K = 2-6) and CV error were shown on the right.
