# Supplementary material for: Exploring the mechanism of artificial selection signature in Chinese indigenous pigs by leveraging multiple bioinformatics database tools
Source: BMC Genomics. 2023 Dec 5;24:743. doi: 10.1186/s12864-023-09848-7 (PMC10699062; doi:10.1186/s12864-023-09848-7)
Supplement: Supplementary file 1 — Additional file 1. Figures S1-S11 and Tables S1-S9. [file 12864_2023_9848_MOESM1_ESM.zip › 02_Supplementary files/Additional file 3_Figure S3_Population genetic structure of Yunnan indigenous pigs and European commercial pigs.pdf]

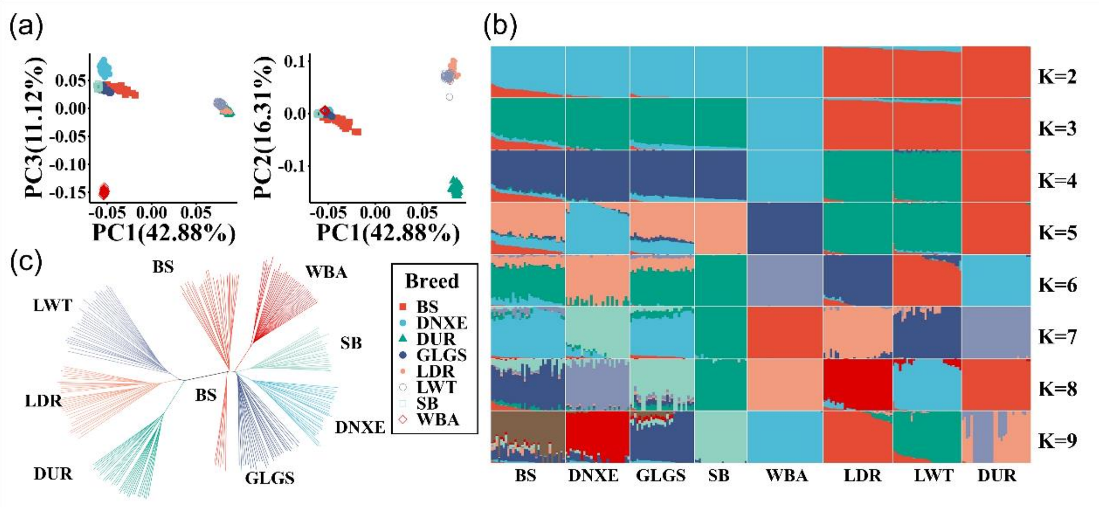

Figure S3 Population genetic structure of Yunnan indigenous pigs, Asian wild boars and European commercial pigs. **a** Principal component analysis (PCA) result of 234 pigs on the first three PCs. **b** Genetic ancestry compositions with the assumed number of ancestries from K=2 to K=9. When K=8, the value of cross validated error was lowest (CV error (K=8): 0.51445). **c** Neighbor-joining phylogenetic tree of 234 pigs. BS, Baoshan pigs; DNXE, Diannanxiaoer pigs; GLGS, Gaoligongshan pigs; SB, Saba pigs; WBA, Asian wild boar; LDR, Landrace pigs; LWT, Large White pigs; DUR, Duroc pigs.
