# Supplementary material for: Exploring the mechanism of artificial selection signature in Chinese indigenous pigs by leveraging multiple bioinformatics database tools
Source: BMC Genomics. 2023 Dec 5;24:743. doi: 10.1186/s12864-023-09848-7 (PMC10699062; doi:10.1186/s12864-023-09848-7)
Supplement: Supplementary file 1 — Additional file 1. Figures S1-S11 and Tables S1-S9. [file 12864_2023_9848_MOESM1_ESM.zip › 02_Supplementary files/Additional file 4_Figure S4_Treemix introgression among Yunnan indigenous pigs.pdf]

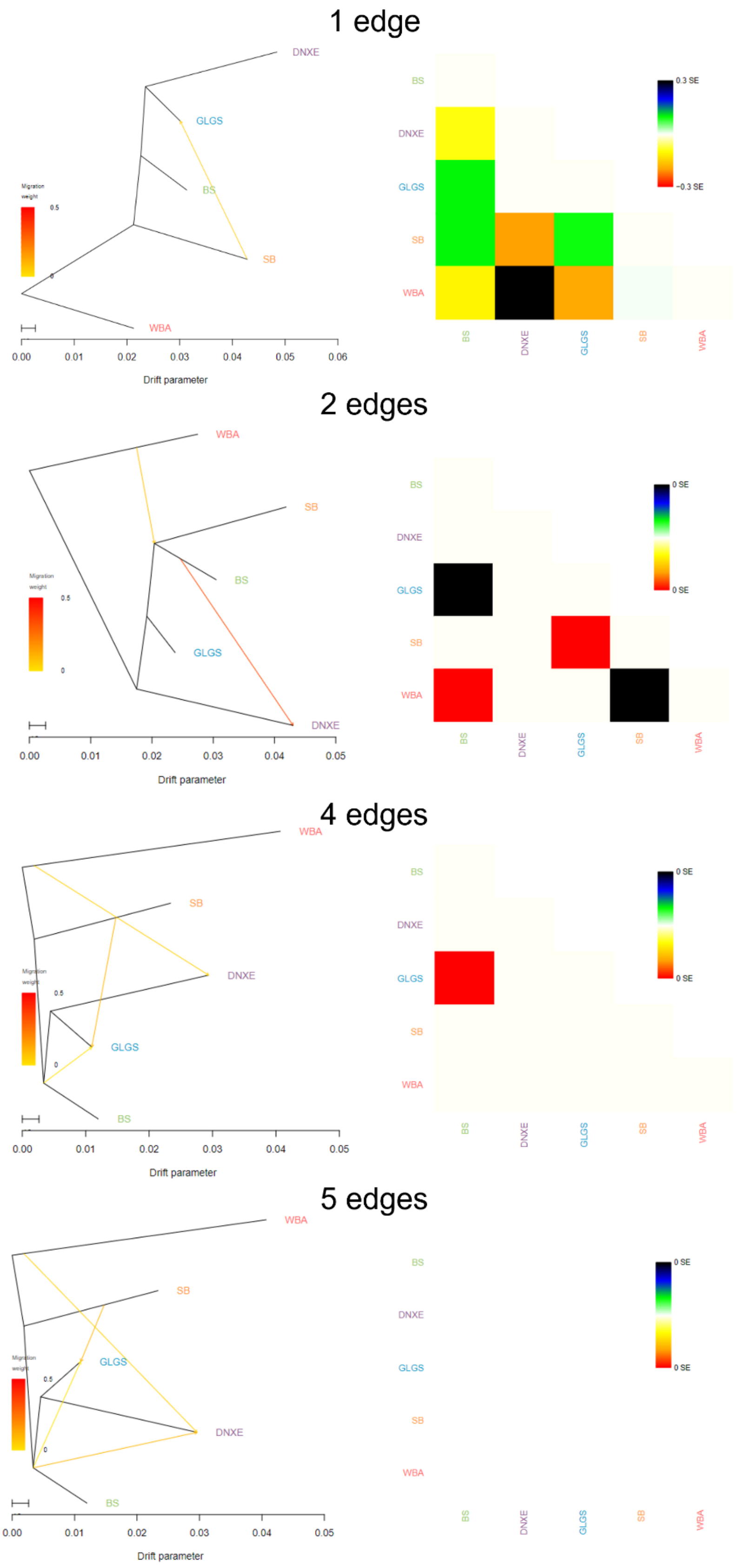

Figure S4 Treemix introgression among the populations and correspondent residence when migration events (m) = 1,2,4,5.
