# Supplementary material for: Exploring the mechanism of artificial selection signature in Chinese indigenous pigs by leveraging multiple bioinformatics database tools
Source: BMC Genomics. 2023 Dec 5;24:743. doi: 10.1186/s12864-023-09848-7 (PMC10699062; doi:10.1186/s12864-023-09848-7)
Supplement: Supplementary file 1 — Additional file 1. Figures S1-S11 and Tables S1-S9. [file 12864_2023_9848_MOESM1_ESM.zip › 02_Supplementary files/Additional file 6_Figure S5_Migration events analysis among Yunnan indigenous pigs, European commerical pigs and WBA.pdf]

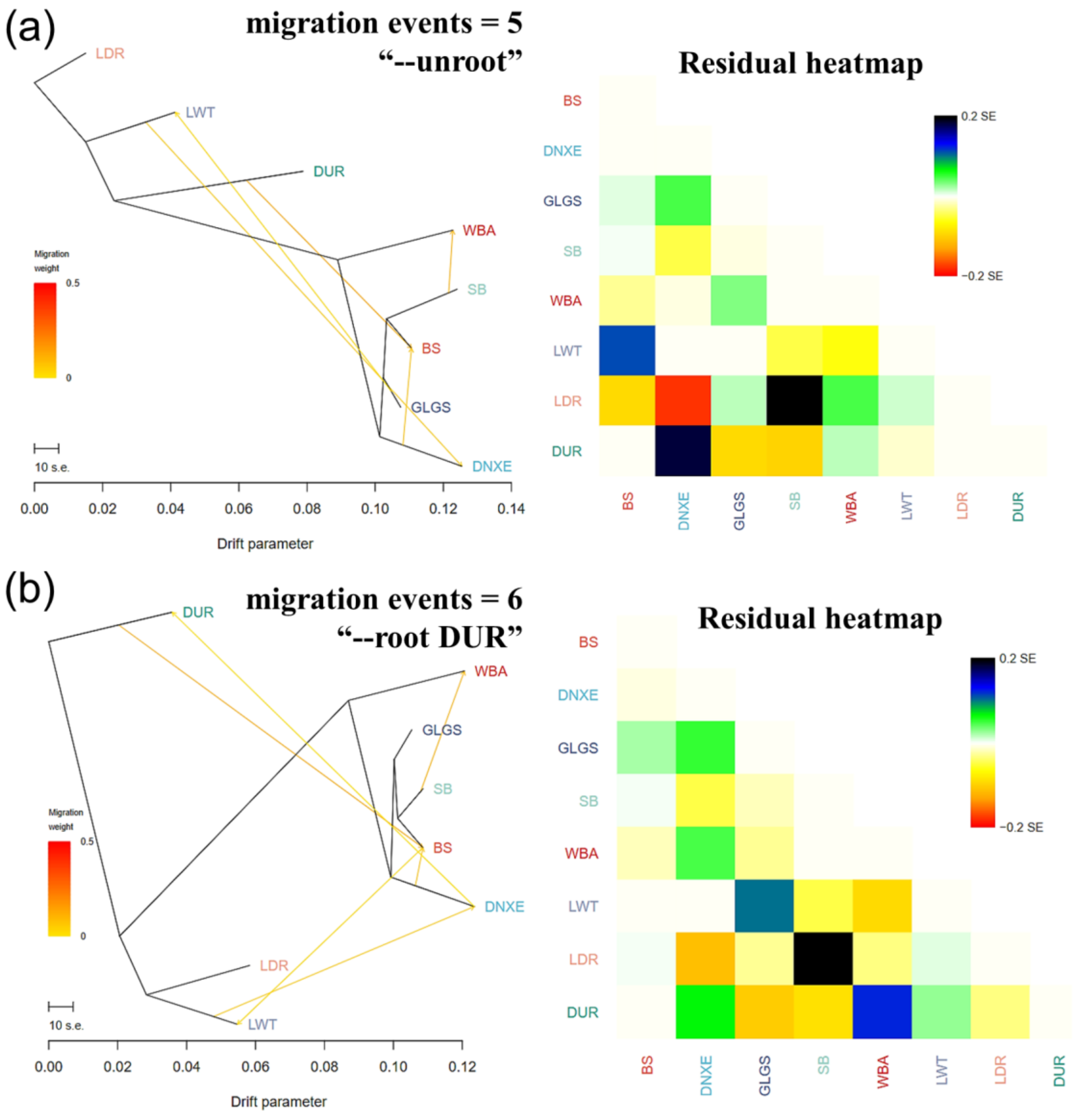

Figure S5 TreeMix migration events extrapolation among Yunnan indigenous pigs, European commercial pigs and Asian wild boars. **a** TreeMix introgression among the populations when m=5 without setting the root group. **b** TreeMix introgression among the populations when m=6 with setting Duroc taxa as the root group. The left panel is the migration tree and the right one is the corresponding residual heatmap. BS, Baoshan pigs; DNXE, Diannanxiaoer pigs; GLGS, Gaoligongshan pigs; SB, Saba pigs; WBA, Asian wild boar; LDR, Landrace pigs; LWT, Large White pigs; DUR, Duroc pigs.
