# Supplementary material for: Exploring the mechanism of artificial selection signature in Chinese indigenous pigs by leveraging multiple bioinformatics database tools
Source: BMC Genomics. 2023 Dec 5;24:743. doi: 10.1186/s12864-023-09848-7 (PMC10699062; doi:10.1186/s12864-023-09848-7)
Supplement: Supplementary file 1 — Additional file 1. Figures S1-S11 and Tables S1-S9. [file 12864_2023_9848_MOESM1_ESM.zip › 02_Supplementary files/Additional file 8_Figure S7_Manhattan plot of selection signatures detected in Yunnan pigs and WBA.pdf]

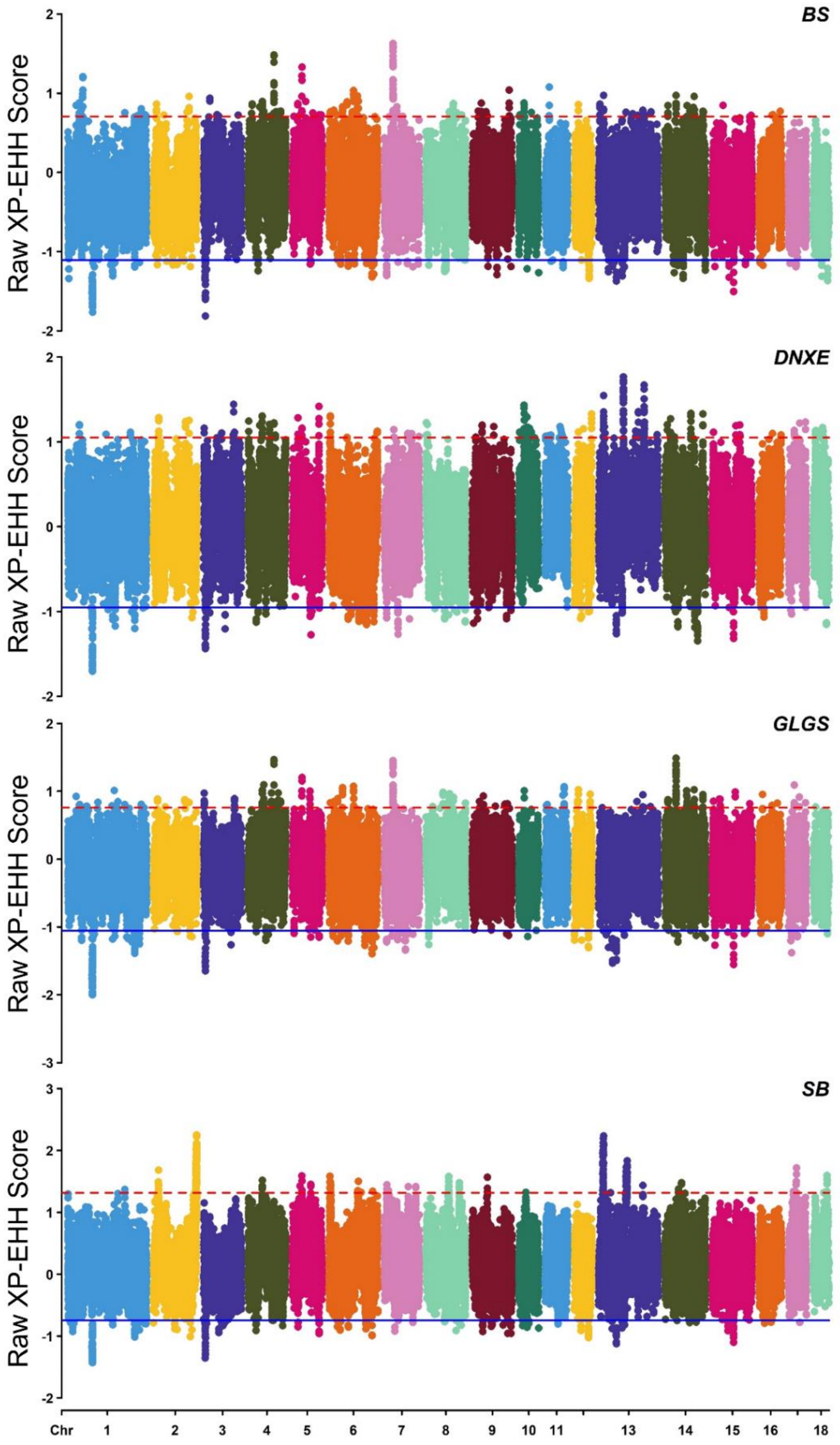

Figure S7 Manhattan plot of selection signatures detected in Yunnan pigs and WBA using XP-EHH method. The *x*-axis and *y*-axis represent chromosome number and the raw XP-EHH score. Lines in red and blue color correspond to the 0.5% and 99.5% threshold.
