# Supplementary material for: Exploring the mechanism of artificial selection signature in Chinese indigenous pigs by leveraging multiple bioinformatics database tools
Source: BMC Genomics. 2023 Dec 5;24:743. doi: 10.1186/s12864-023-09848-7 (PMC10699062; doi:10.1186/s12864-023-09848-7)
Supplement: Supplementary file 1 — Additional file 1. Figures S1-S11 and Tables S1-S9. [file Additional file 20_Figure S11_Gene expression and associated phenotypes regulated by CCT2, FRS2.pdf › 02_Supplementary files/Additional file 20_Figure S11_Gene expression and associated phenotypes regulated by CCT2, FRS2.pdf]

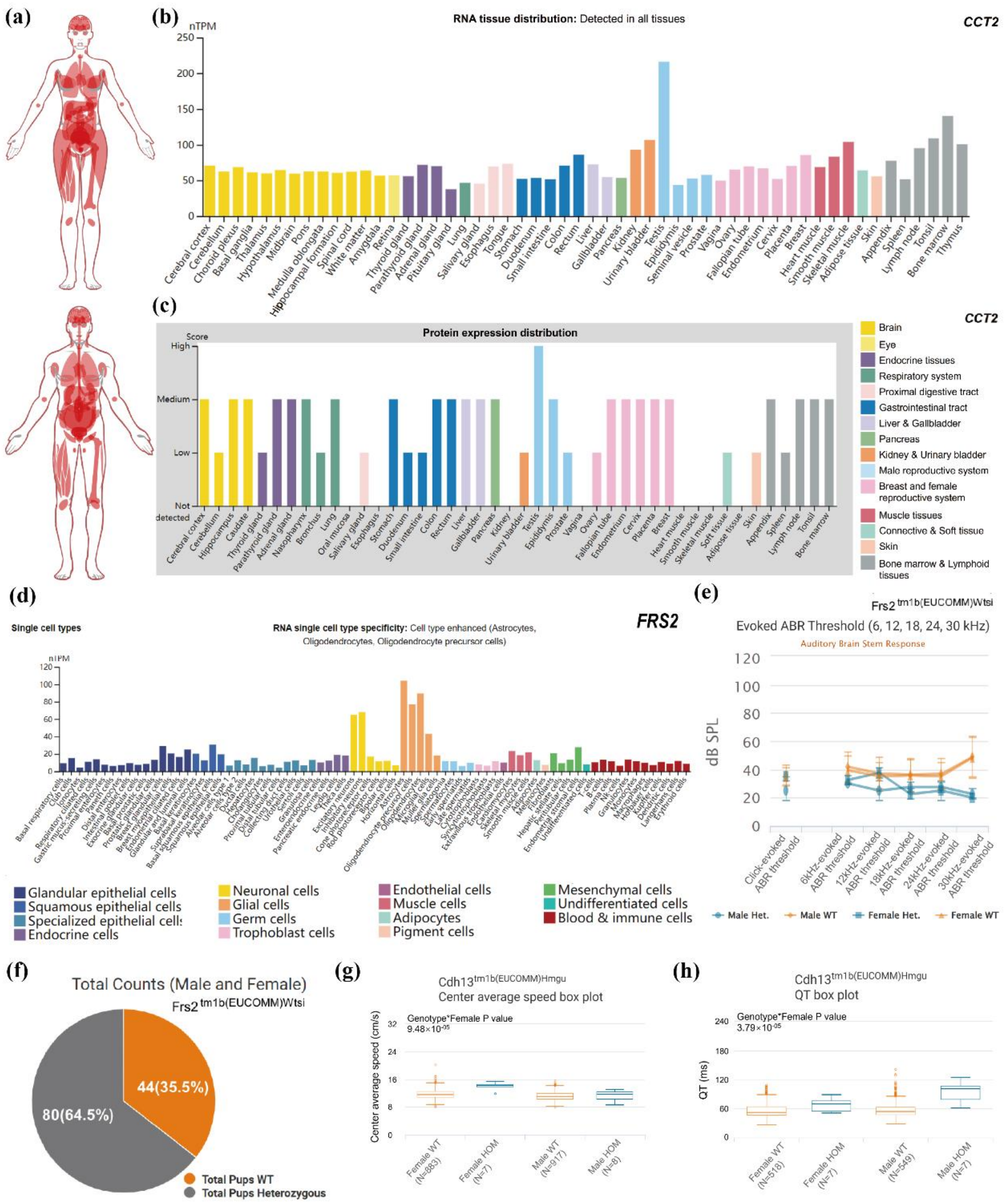

Figure S11 Gene expression and associated phenotypes regulated by *CCT2*, *FRS2* and *CDH13* in IMPC and HPA databases. **a-c** Human tissue distributions of *CCT2* RNA expression and protein expression from HPA. **d** *FRS2* RNA single cell type expression had specificity in glial cells and neuronal cells. **e** *Frs2*$^{tm1b(EUCOMM)Wtsi}$ hetegyzosity mutant mice showed abnormal auditory brain stem response through measuring 30kHz-evoked ABR threshold in 2 female, 2 male mutants compared to 196 female, 205 male controls (*P*=1.35E-5). **f** Proportion of wild type, heterozygous, and homozygous offspring of a mutant mice strain carrying the Frs2$^{tm1b(EUCOMM)Wtsi}$ allele. **g** *CDH13* was associated to hyperactivity phenotype in mice, which was assayed by measuring Center average speed in 7 female, 8 male mutants compared to 891 female, 919 male controls. The mutants are for the *Cdh13*$^{tm1b(EUCOMM)Hmgu}$ allele (*P*=9.33E-5). **h** *Cdh13*$^{tm1b(EUCOMM)Hmgu}$ homogyzosity mutant mice showed shortened QT interval through measuring QT in 7 female, 7 male mutants compared to 518 female, 549 male controls (*P*=3.79E-5).
